# A Cross-Reactive Small Protein Binding Domain Provides a Model to Study Off-Tumor CAR-T Cell Toxicity

**DOI:** 10.1016/j.omto.2020.04.001

**Published:** 2020-04-14

**Authors:** Joanne A. Hammill, Jacek M. Kwiecien, Anna Dvorkin-Gheva, Vivian W.C. Lau, Christopher Baker, Ying Wu, Ksenia Bezverbnaya, Craig Aarts, Christopher W. Heslen, Galina F. Denisova, Heather Derocher, Katy Milne, Brad H. Nelson, Jonathan L. Bramson

**Affiliations:** 1 Department of Pathology and Molecular Medicine, McMaster University, Hamilton, ON L8S 4K1, Canada; 2 Deeley Research Centre, BC Cancer, Victoria, BC V8R 6V5, Canada

**Keywords:** cell therapy, chimeric antigen receptor, CAR-T cell, xenograft model, off-tumor toxicity

## Abstract

Tumor-targeted chimeric antigen receptor (CAR)-engineered T lymphocytes (CAR-T cells) have demonstrated striking clinical success, but their use has been associated with a constellation of toxicities. A better understanding of the pathogenesis of these toxicities is required to improve the safety profile of CAR-T cells. Herein, we describe a xenograft model of off-tumor CAR-T cell-associated toxicity. Human CAR-T cells targeted against HER2 using a small-protein binding domain induced acute, dose-dependent toxicities in mice. The inclusion of a CD28 or 4-1BB co-stimulatory domain in the CAR was required to produce toxicity; however, co-stimulation through CD28 was most toxic on a per-cell basis. CAR-T cell activation in the lungs and heart was associated with a systemic cytokine storm. The severity of observed toxicities was dependent upon the peripheral blood mononuclear cell (PBMC) donor used as a T cell source and paralleled the CD4^+^-to-CD8^+^ T cell ratio in the adoptive transfer product. CD4^+^ CAR-T cells were determined to be the primary contributors to CAR-T cell-associated toxicity. However, donor-specific differences persisted after infusion of a purified CD4^+^ CAR-T cell product, indicating a role for additional variables. This work highlights the contributions of CAR-T cell-intrinsic variables to the pathogenesis of off-tumor toxicity.

## Graphical Abstract

**Figure:**
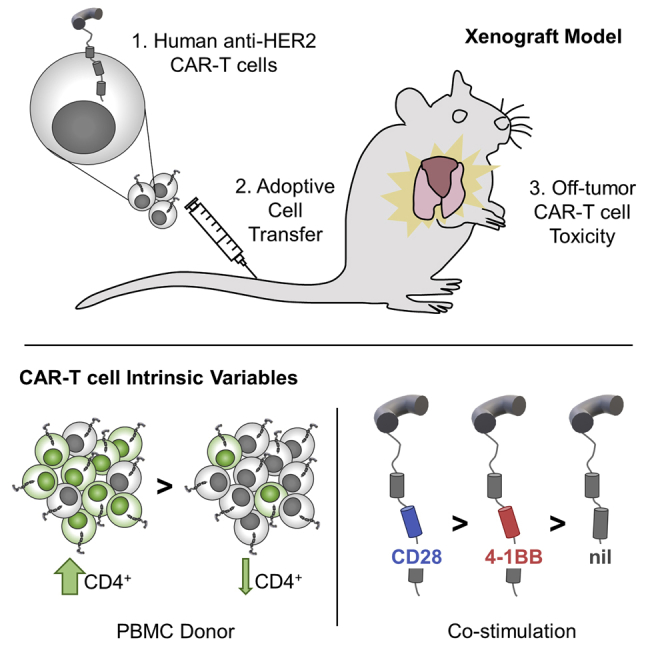

## Teaser

Chimeric antigen receptor-engineered T cells (CAR-T cells) are capable of striking anti-tumor efficacy. However, their use has been associated with a constellation of toxicities. Herein, Bramson and colleagues describe a xenograft model of off-tumor CAR-T cell toxicity in a murine host. This model is used to investigate how variables intrinsic to a human CAR-T cell product, including PBMC donor, contribute to severity of toxicity.

## Introduction

The adoptive transfer of chimeric antigen receptor (CAR)-engineered T lymphocytes (CAR-T cells) for the treatment of cancer has generated striking clinical success.^1^, ^2^, ^3^, ^4^, ^5^, ^6^, ^7^ This success has been paralleled by a constellation of CAR-T cell-associated toxicities, ranging in severity from mild to life threatening, of which the pathogenesis is incompletely understood.^8^, ^9^, ^10^ Better understanding of the factors contributing to CAR-T cell toxicities is critical for the development of therapeutics with an improved safety profile.

CARs, as reviewed by Jackson et al.^11^ and by June and Sadelain,^12^ are recombinant proteins that, when engineered for expression on the surface of T lymphocytes, redirect those T cells against a tumor target. CARs are composed of an extracellular antigen recognition domain, specific for a tumor target, and intracellular T cell activation domains, which trigger T cell effector functions and cytotoxicity upon target ligation. Second-generation CARs, which dominate the clinic, pair an intracellular T cell activation signal (primarily CD3ζ) with a co-stimulatory domain (typically either CD28 or 4-1BB). Currently, most CAR-T cells are prepared as an autologous product where the patient’s own T cells are extracted, engineered to express the CAR, and infused into the patient as a cellular drug.

CAR-T cell-associated toxicities can be broadly classified into cytokine-associated and autoimmune toxicities,^13^ although these categories are not mutually exclusive. Cytokine-associated toxicities—most commonly, cytokine release syndrome (CRS) and immune effector cell-associated neurotoxicity syndrome (ICANS)—arise due to the elevation in systemic levels of inflammatory cytokines resulting from robust CAR-T cell activation.^8^^,^^9^^,^^14^^,^^15^ Autoimmune toxicities arise when CAR-T cells respond against healthy, non-tumor tissues.

“On-target, off-tumor” autoimmune toxicity occurs when CAR-T cells respond against their target antigen on non-tumor tissue. Clinical use of CAR-T cell therapies for hematological tumors has been associated with the destruction of non-tumor tissues, as targets like CD19 and BCMA are expressed on healthy B cells and plasma cells.^16^^,^^17^ However, these cells are considered non-essential, as their loss can be managed by intravenous immunoglobulin therapy. On-target, off-tumor toxicities against essential tissues arising during the treatment of solid tumors have had lethal consequences.^18^, ^19^, ^20^ "Off-target, off-tumor" toxicity, caused by the cross-reactivity of CAR-T cells against a non-target antigen, could also theoretically damage healthy tissues,^10^ as has been observed with T cell-receptor (TCR)-engineered T cells.^21^ Autoimmune toxicities can be managed by selecting targets unique to the tumor and performing in-depth cross-reactivity analysis to ensure that the CAR is antigen specific. However, as CAR-T cell therapy expands into the realm of solid tumors, where many targetable tumor antigens are expressed at low levels on healthy tissues, off-tumor toxicities are likely to become more prevalent. Therefore, to mitigate severe adverse events, it is imperative that we understand the features of CAR-T cell products that influence autoimmune toxicities.

Here, we describe a pre-clinical xenograft model of off-tumor toxicity where efficacy of the CAR-T cell therapy was associated with severe, often lethal, toxicities. While CARs bearing either CD28 or 4-1BB co-stimulatory domains were capable of triggering toxicity, CD28-bearing CARs were more potent. Off-tumor, off-target activation of CAR-T cells in the lungs and heart caused a systemic cytokine storm. We observed differences in toxicity onset and severity dependent upon the peripheral blood mononuclear cell (PBMC) donor used to generate the CAR-T cell product and attributed these differences, in part, to the frequency of CD4^+^ T cells in the cell product. However, even products generated from purified CD4^+^ T cells exhibited donor-specific differences; these correlated with *in vivo* expansion and cytokine production. These data highlight how intrinsic properties of the CAR-T cell product can contribute to off-tumor toxicity.

## Results

### Second-Generation DARPin-Targeted Anti-HER2 CAR-T Cells Were Toxic *In Vivo*

**Figure 1:**
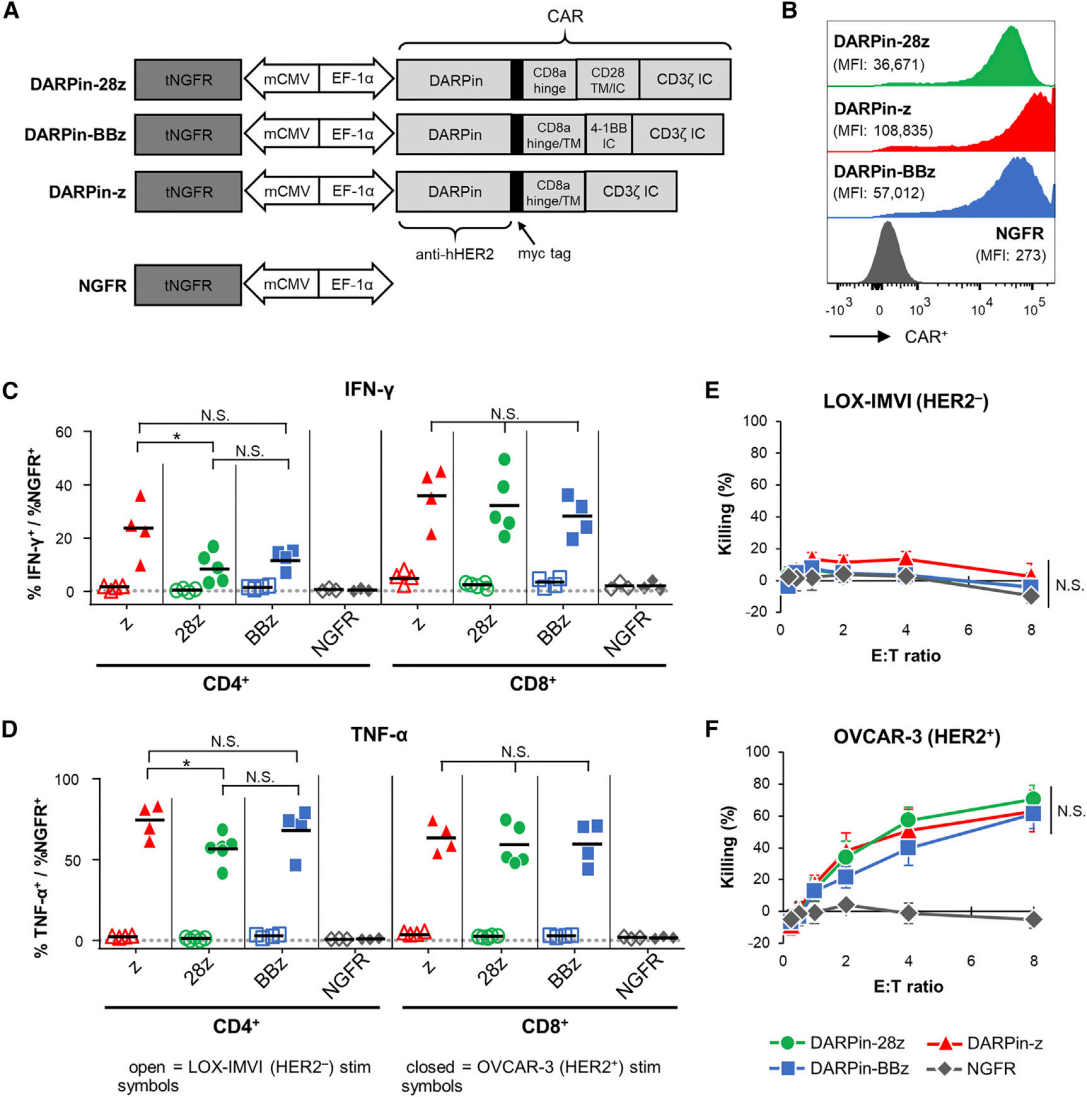
Anti-HER2 DARPin-Targeted CAR-T Cells Demonstrated Similarl Efficacy *In Vitro* (A) Schematics of the dual-promoter lentiviral (LV) gene cassettes used to generate anti-HER2 DARPin-targeted first- or second-generation CAR-T cells (structural details are as indicated; TM, transmembrane; IC, intracellular) or CAR-negative control NGFR-T cells. In all cases, truncated NGFR (tNGFR) is included as a transduction marker. (B) Expression of CARs on the surface of engineered (NGFR^+^) T cells as determined by flow cytometry (upstream gating strategy: lymphocytes → singlets → NGFR^+^). Mean fluorescence intensity (MFI) for CAR expression is indicated in brackets. Representative results have been replicated in 2–4 additional independent experiments. (C and D) Production of IFN-γ (C) and TNF-α (D) upon CAR-T cell stimulation with HER2^+^ (OVCAR-3; closed symbols) or HER2^−^ (LOX-IMVI; open symbols) human tumor cell lines was measured by intracellular cytokine staining (ICS) and subsequent flow cytometry (upstream gating strategy: lymphocytes → singlets → CD4^+^ or CD8^+^ T cells). Percent cytokine production was normalized for transduction (transduction ranges observed: DARPin-28z, 39%–60%; DARPin-BBz, 33%–52%; DARPin-z, 25%–63%; NGFR, 63%–86%). Each point indicates data from a single independent experiment (n = 3–5 per LV construct); black lines indicate mean values. (E and F) Cytotoxicity across various effector:target (E:T) ratios with LOX-IMVI (E) or OVCAR-3 (F) tumor cell targets; ratios are based on total T cell numbers and have not been normalized for transduction. Error bars represent standard error of the mean (SEM). Data are from n = x independent experiments, as follows: DARPin-z, 4; DARPin-28z, 5; DARPin-BBz, 4; NGFR, 3.

Primary human T cells were engineered with a variety of CARs targeted against HER2 using a designed ankyrin repeat protein (DARPin) (Figure 1A): (1) a CAR containing the intracellular signaling domains from CD3ζ and CD28 (DARPin-28z, as reported by Hammill et al.^22^); (2) a CAR containing the intracellular signaling domains from CD3ζ and 4-1BB (DARPin-BBz); or (3) a CAR containing CD3ζ alone (DARPin-z). As a negative control, T cells were engineered with a lentivirus encoding truncated low-affinity nerve growth factor receptor (NGFR) alone (NGFR-T cells) (Figure 1A). All three CARs were similarly expressed on the surface of engineered primary human T cells (Figure 1B). Upon stimulation with a HER2-positive tumor cell line (OVCAR-3), DARPin-28z-, DARPin-BBz-, and DARPin-z-T cells all showed a similar capacity to produce interferon (IFN)-γ (Figure 1C, closed symbols) and tumor necrosis factor alpha (TNF-α) (Figure 1D, closed symbols); these CAR-T cells were not stimulated by the HER-2-negative line, LOX-IMVI (Figures 1C and 1D, open symbols). All three DARPin-targeted CAR-T cells were similarly cytotoxic against OVCAR-3 tumor cells while sparing LOX-IMVI tumor cells (Figures 1E and 1F). NGFR-T cells were functionally unresponsive against either tumor cell line.

**Figure 2:**
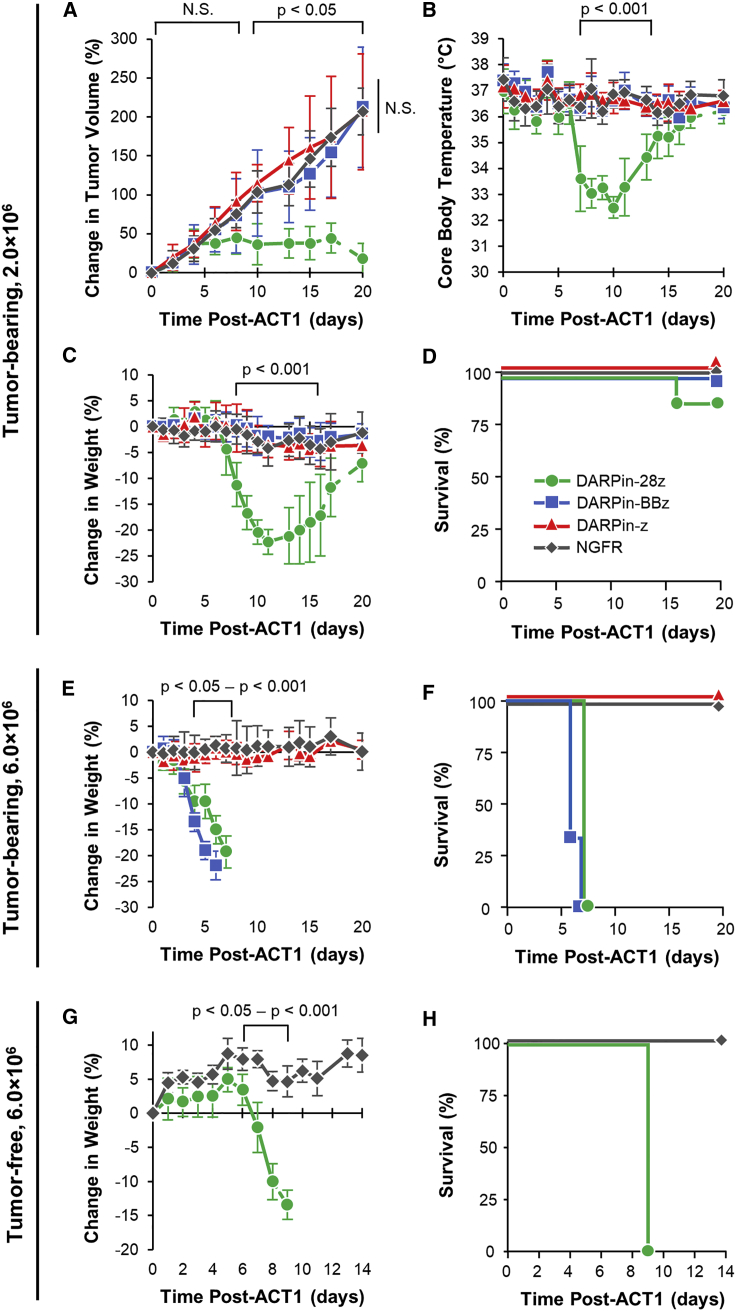
DARPin-28z-T Cells Demonstrated Dose-Dependent, Lethal Off-Tumor Toxicity *In Vivo* (A–D) OVCAR-3 tumor-bearing NRG mice were treated with 2.0 × 10^6^ engineered T cells (DARPin-28z, green circles; DARPin-BBz, blue squares; or DARPin-z, red triangles, as indicated) or an equal or greater number of NGFR-T cells (gray diamonds). Mice were monitored over time for tumor volume (A), core body temperature (B), weight (C), and survival (D). Data are pooled from two independent experiments; n = 8 for CAR groups; n = 7 for the NGFR group. Lines become dashed after the first mouse in the group succumbs to toxicity. Error bars indicate standard deviation (SD). (E and F) OVCAR-3 tumor-bearing NRG mice were treated with 6.0 × 10^6^ DARPin-28z-T cells or an equal or greater number of NGFR-T cells. Mice were monitored over time for weight (E) and survival (F). Data are pooled from one to two independent experiments with n = x mice per treatment: DARPin-28z, 7; DARPin-BBz, 3; DARPin-z, 4; NGFR, 4. (G and H) Tumor-free NRG mice were treated with 6.0 × 10^6^ DARPin-28z-T cells or an equal or greater number of NGFR-T cells. Mice were monitored over time for weight (G) and survival (H). Representative data from 1 experiment are shown (DARPin-28z, n = 3; NGFR, n = 4); DARPin-28z toxicity in tumor-free mice has been observed in seven additional independent experiments. In all cases, error bars represent SD. The p values are as indicated; N.S., not significant.

To evaluate whether differences in efficacy would manifest *in vivo*, NRG mice bearing subcutaneous OVCAR-3 tumors were treated with 2.0 × 10^6^ CAR-T cells. Despite displaying similar effector function *in vitro*, only DARPin-28z-T cells demonstrated anti-tumor efficacy *in vivo*; tumor growth in DARPin-BBz- and DARPin-z-T cell-treated mice was no different than that in NGFR-T cell-treated controls (Figure 2A). Severe toxicity was observed after DARPin-28z-T cell treatment. Symptoms of toxicity included decreased body condition, hunched posture, ruffled coat, and labored breathing; simultaneous decreases in core body temperature (Figure 2B) and weight loss (Figure 2C) were used as quantifiable measures of toxicity onset and severity. Toxicity was lethal in 1 of 8 mice within 20 days of DARPin-28z-T cell treatment (Figure 2D). The DARPin-BBz- and DARPin-z-T cells did not reveal toxicities at this dose.

Escalating the T cell dose to 6.0 × 10^6^ CAR-T cells per mouse revealed toxicities for both DARPin-28z- and DARPin-BBz-T cells. The onset and severity of toxicity by both second-generation CARs was much more rapid, demonstrating dose dependence (Figures 2E and 2F). Importantly, mice treated with DARPin-z- or NGFR-T cells showed no evidence of toxicity at either dose level tested, emphasizing the importance of co-stimulation in the pathogenesis of toxicity. Lowering the dose of CAR-T cells to 0.66 × 10^6^ DARPin-28z-T cells per mouse attenuated the toxic profile but also blunted the anti-tumor efficacy (Figure S1), suggesting that the two events were linked. Similar levels of toxicity were observed when tumor-free mice were treated with DARPin-28z-T cells, indicating that the observed toxicity resulted from an attack against healthy tissues (off-tumor) (Figures 2G and 2H).

Of the 3 types of CAR-T cells tested in these experiments, the DARPin-28z-T cells displayed the highest functional avidity, which may explain why these CAR-T cells yielded the most robust anti-tumor activity and toxicity profile (Figure S2). However, it remains unclear whether differences in functional activity are a driving force influencing *in vivo* effects, as the DARPin-BBz- and DARPin-z-T cells displayed a similar functional avidity (Figure S2), even though DARPin-BBz-T cells produced greater toxicity *in vivo*.

### DARPin-28z-T Cells Became Activated in Pulmonary and Cardiac Tissues, Resulting in a Systemic Cytokine Storm

To determine the site of off-tumor toxicity, total body necropsies were performed on DARPin-28z- or NGFR-T cell-treated mice. Tissues were interrogated by hematoxylin and eosin (H&E) and immunohistochemistry (IHC) for human CD3, and analyzed in a blinded fashion by a veterinary pathologist. Aberrant immune cell infiltration of pulmonary and cardiac tissues was reproducibly observed in DARPin-28z-T cell-treated mice; no such infiltration was found in matched NGFR-T cell-treated counterparts. Other tissues showed only a scattered presence of CD3^+^ cells, which were not associated with any pathology and were similar between DARPin-28z- and NGFR-T cell-treated mice.

**Figure 3:**
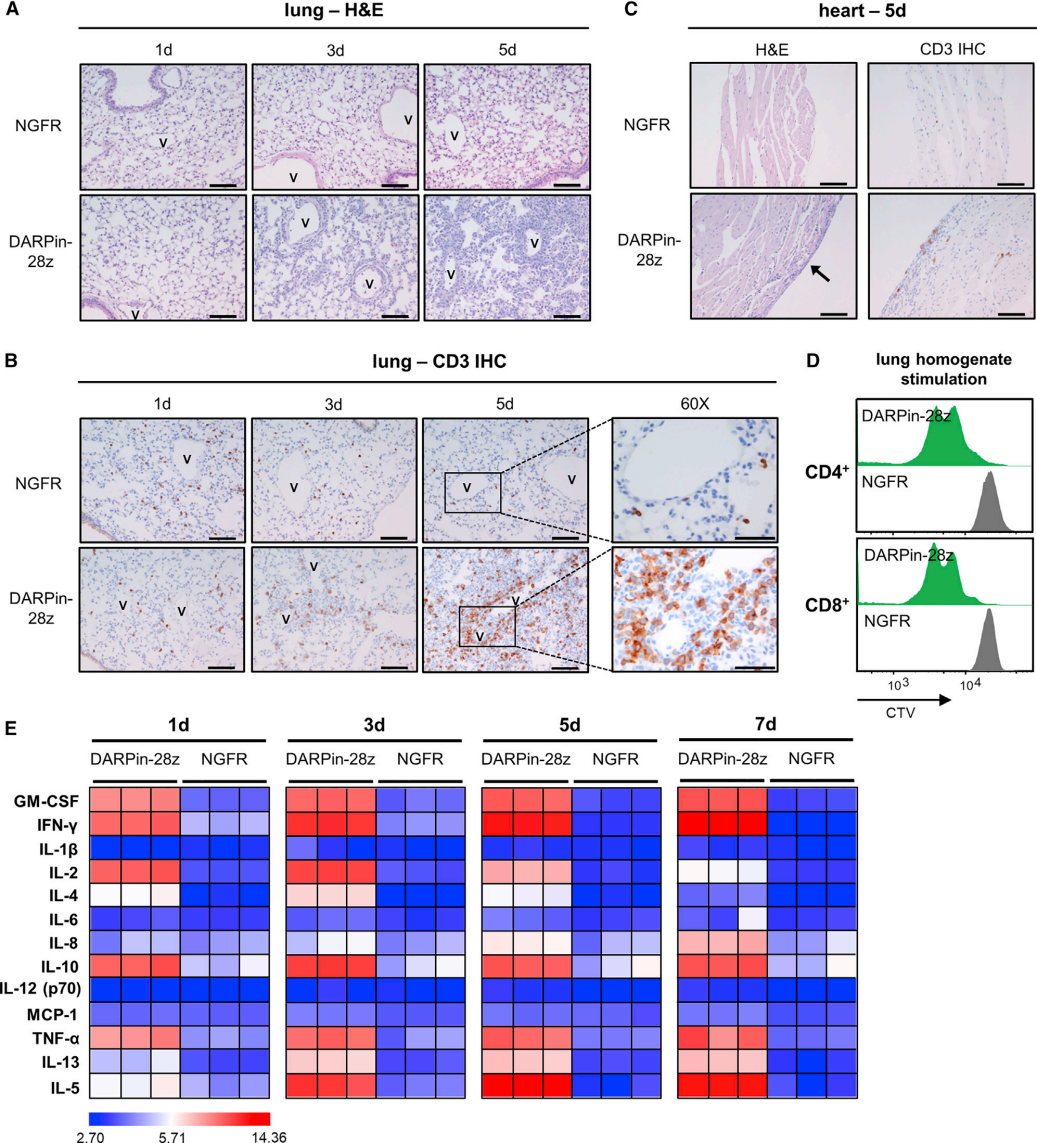
DAPRin-28z-T Cells Activated in the Lungs and Heart, Resulting in a Systemic Cytokine Storm OVCAR-3 tumor-bearing NRG mice were treated with 6 × 10^6^ effective DARPin-28z-T cells or a matched number of NGFR-T cells. (A–C) Mice were sacrificed at 1, 3, or 5 days post-ACT1 for total body perfusion, fixation, necropsy, and histological analysis. (A) Hematoxylin and eosin (H&E) staining of the lungs at 20× magnification (scale bars, 100 μm). V, vasculature. (B) Immunohistochemistry (IHC) for human CD3 in the lungs at 20× magnification (scale bars, 100 μm) or 60× magnification (zoom-in; scale bars, 50 μm). (C) H&E or CD3 IHC staining of the heart at 20× magnification (scale bars, 100 μm); arrow indicates aberrant region of inflammation along the right heart wall. Representative images from n = 2–3 mice are shown. Findings have been recapitulated in 1–2 additional independent experiments. (D) DARPin-28z- or NGFR-T cells were co-cultured with tumor-free NRG mouse lung homogenates *ex vivo*. T cell proliferation was measured by flow cytometry using CellTrace Violet (CTV) dye. Data are representative of two independent experiments. (E) Mice were bled at 1, 3, 5, or 7 days post-ACT1 for multiplex analysis of human serum cytokine content; a globally normalized heatmap of log2-transformed human cytokine fluorescence readings is shown. Each square represents data from one mouse. Colorimetric scale bar indicates minimum, average, and maximum values on map. Absolute values are displayed in Table S1. Results are consistent with those observed in two additional independent experiments. Murine cytokine levels from the same mice are presented in Figure S5 and Table S2.

Pulmonary immune infiltrate in DARPin-28z-T cell-treated mice, as observed with H&E, began forming at subpleural areas and at perivascular cuffs as early as 1 day post-adoptive cell transfer dose 1 (ACT1), becoming more severe over time (Figure 3A). The infiltrate contained scattered neutrophils. IHC for human CD3 confirmed the concentrated presence of human T cells, and cause of death was attributed to pneumonitis. In contrast, NGFR-T cell-treated mice had only scattered T cells throughout the lungs. Furthermore, pulmonary DAPRin-28z T cells were much larger than matched NGFR-T cells, suggesting that DARPin-28z-T cells were activated (Figure 3B).

In the myocardium, moderate, patchy immune deposits formed in the papillary muscle or the right heart wall of DARPin-28z-T cell-treated mice, starting at 3 days post-ACT1 and reaching ubiquity (3/3 mice) by 5 days post-ACT1. NGFR-T cell-treated mice only rarely showed small, scattered T cells in cardiac tissue (Figure 3C).

*Ex vivo*, DARPin-28z-T cells, unlike NGFR-T cell controls, proliferated in response to stimulation with lung homogenates from tumor-free NRG mice (Figure 3D), confirming the off-tumor, acute nature of the activation. DARPin-28z-T cells did not respond to stimulation with a murine HER2 (mHER2)-engineered cell line, indicating that the toxicity was not the result of cross-reactivity against mHER but, rather, an off-target response (Figure S3). While the exact antigenic target remains unknown, stimulation of DARPin-28z-T cells with a panel of tissue extracts supported necropsy findings and suggested that the antigen has tissue-restricted expression; DARPin-28z-T cells proliferated most robustly following stimulation with lung and heart homogenates, whereas the proliferation of DARPin-28z-T cells was weak, or absent, when stimulated with lysates from other tissues (Figure S4).

Multiplex analysis of human cytokines in the serum of the treated mice revealed a marked cytokine storm resulting from the activation of the DARPin-28z-T cells, which exacerbated over time (Figure 3E; Figure S5; Tables S1 and S2), and this toxicity could be mitigated by corticosteroid treatment (Figure S6).

### DARPin-28z-T Cell Toxicity Was Donor Dependent

**Figure 4:**
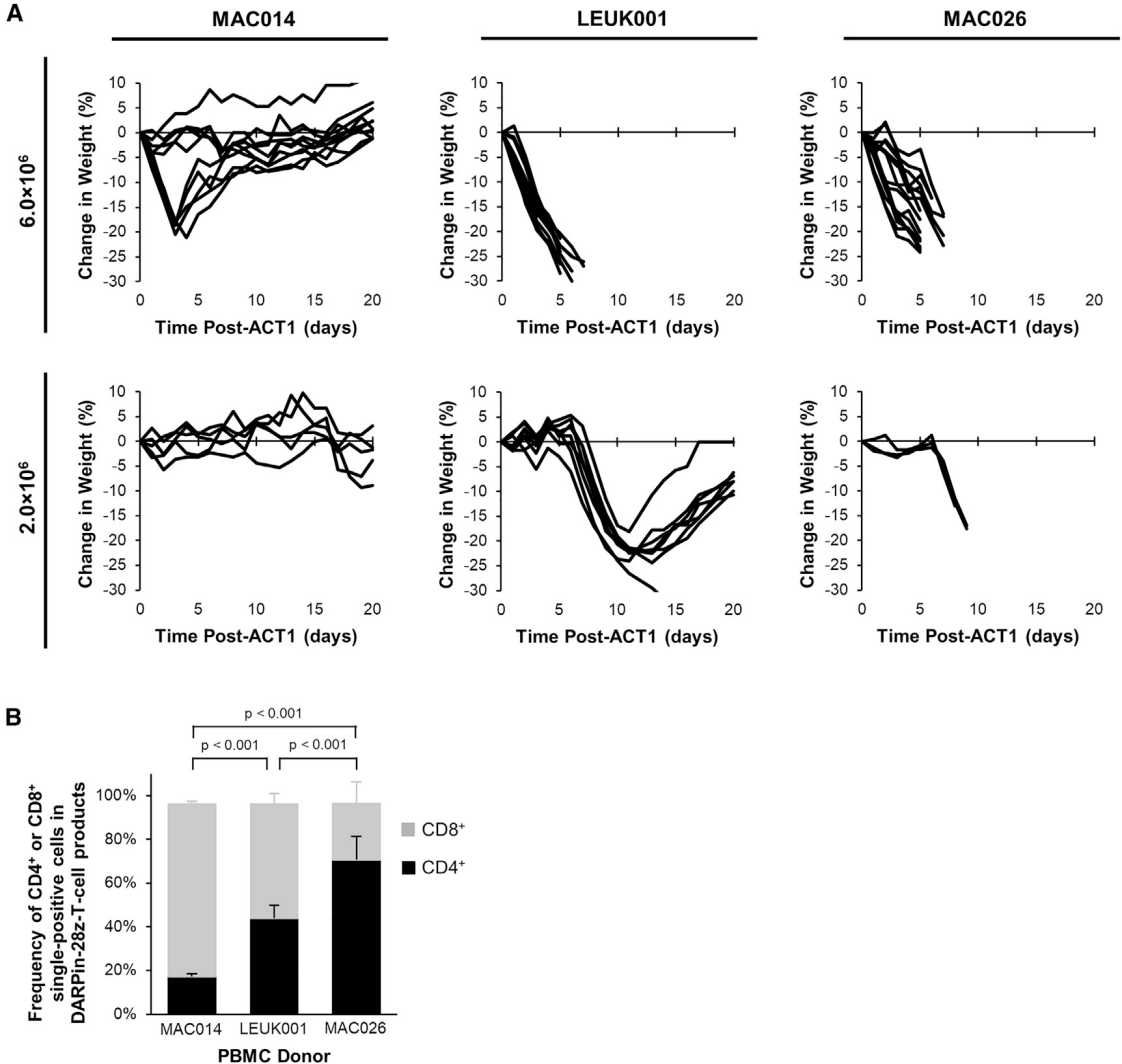
Differential *In Vivo* Toxicity of DARPin-28z-T Cells Manufactured from Unique PBMC Donors Correlated with the Frequency of CD4+ T Cells in the Adoptive Transfer Product (A) OVCAR-3 tumor-bearing NRG mice were treated with 6.0 × 10^6^ or 1.7–2.0 × 10^6^ DARPin-28z-T cells produced from MAC026, LEUK001, or MAC014 PBMCs. Mice were monitored over time for changes in weight. Data were pooled from n = x independent experiments. For 6.0 × 10^6^ cells, MAC014, 2; LEUK001, 3; and MAC026, 4. For 2.0 × 10^6^ cells, MAC014, 1; LEUK001, 2; and MAC026, 1. Each line indicates data from one animal; curves end, indicating when mice succumbed to toxicity. (B) Composition of CD4^+^ or CD8^+^ cells in DARPin-28z-T cell products (days 13–14 post-activation) manufactured using starting PBMCs from donors as indicated and determined using flow cytometry (upstream gating strategy: lymphocytes → singlets → NGFR^+^). Error bars represent SD. Data from n = x independent experiments; MAC014, 5 (2 unique PBMC preparations); LEUK001, 6 (1 PBMC preparation); and MAC026, 12 (5 unique PBMC preparations).

To begin understanding the factors that influence toxicity, NRG mice bearing OVCAR-3 tumors were treated with 6.0 × 10^6^ or 2.0 × 10^6^ DARPin-28z-T cells manufactured from three different PBMC donors (MAC026, LEUK001, and MAC014). The donor-variant DARPin-28z-T cell products displayed dramatically different properties *in vivo*. MAC014-derived DARPin-28z-T cells produced a mild, transient toxicity at the 6.0 × 10^6^ dose and revealed no toxicities at the 2.0 × 10^6^ dose (Figure 4A). In contrast, both MAC026- and LEUK001-derived DARPin-28z-T cells produced extreme toxicities. At the 6.0 × 10^6^ dose, DARPin-28z-T cells derived from either donor displayed lethal toxicity (Figure 4A). At the 2.0 × 10^6^ dose, despite the equivalent onset of toxicity, only mice treated with LEUK001-derived DAPRin-28z T cells were able to recover (Figure 4A). Our standard *in vitro* analysis of these T cell products (Figures S7A–S7C) had not predicted the observed MAC014 < LEUK001 < MAC026 hierarchy of toxicity *in vivo*; DARPin-28z-T cell products from all three donors showed similar CAR expression and produced similar levels of activation cytokines upon stimulation *in vitro*. MAC014 DARPin-28z-T cells showed the greatest cytotoxicity against HER2^+^ tumor cell targets *in vitro* (Figures S7D and S7E). The only *in vitro* characteristic of the donor-variant DARPin-28z-T cell products that correlated with toxicity was the frequency of CD4^+^ T cells in the adoptive transfer product (Figure 4B), where MAC014 < LEUK001 < MAC026.

### CD4^+^ T Cells in the DARPin-28z-T Cell Product Were the Critical Drivers of Toxicity

Given the correlation between the frequency of CD4^+^ T cells in the DARPin-28z adoptive transfer product and the severity of toxicity *in vivo*, we hypothesized that CD4^+^ T cells were the critical drivers of toxicity.

**Figure 5:**
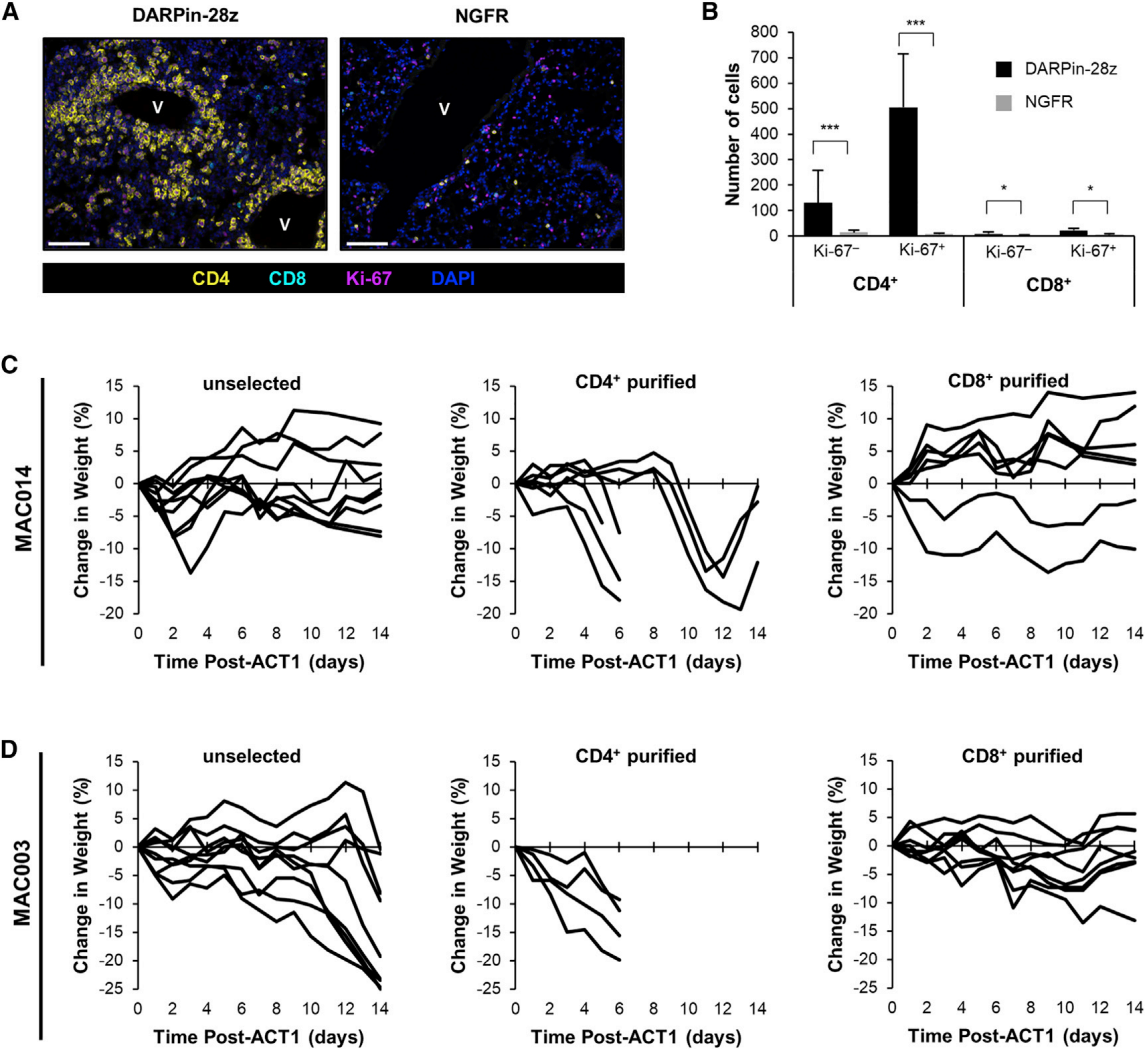
CD4^+^ DARPin-28z-T Cells Were the Primary Drivers of Toxicity (A) 7 days post-ACT1, FFPE lung tissues from DARPin-28z- or NGFR-T cell-treated mice (as described in Figure 2) were analyzed by multiplex immunofluorescence. Slides were stained for CD8 (cyan), CD4 (yellow), DNA (DAPI, blue) and a proliferation marker (Ki-67, magenta). Representative images are from 3 mice; n = 3 images per mouse (scale bars, 100 μm) are shown. (B) Quantification of the full dataset (error bars represent SD). (C and D) OVCAR-3 tumor-bearing or tumor-free NRG mice were treated with DARPin-28z-T cells generated from CD4^+^ purified, CD8^+^ purified, or unselected MAC014 PBMCs (C) (3.2 × 10^6^–6.0 × 10^6^ DARPin-28z-T cells per mouse) or MAC003 PBMCs (D) (6.0 × 10^6^ DARPin-28z-T cells per mouse). Mice were followed for changes in weight; each line indicates data from one mouse; curves end, indicating when mice succumbed to toxicity.

Multiplex immunofluorescence was performed to characterize T cells within the pulmonary immune infiltrate observed in DARPin-28z-T cell-treated mice. Lung slides were stained concurrently for CD4^+^, CD8^+^, and the proliferative marker Ki-67. The pulmonary infiltrate in DARPin-28z-T cell-treated mice was almost entirely composed of Ki-67^+^ CD4^+^ cells, supporting a role for the local proliferation of CD4^+^ CAR-T cells in the pathogenesis of toxicity (Figures 5A and 5B).

To address this hypothesis, we generated T cell products from the least toxic donor, MAC014, using either unselected PBMCs, purified CD4^+^ T cells, or purified CD8^+^ T cells (Figure S8). The T cell products were used to treat OVCAR-3 tumor-bearing or tumor-free mice. Consistent with our previous results, MAC014-derived DARPin-28z-T cells generated from unselected PBMCs remained non-lethal. The product generated from CD8^+^ purified T cells was also non-toxic. In contrast, mice treated with CD4^+^ purified MAC014-derived DAPRin-28z T cells experienced weight loss of up to 20%, and 4/7 mice died within 14 days of treatment (Figure 5C). The same trend was observed with DARPin-28z-T cells generated from a second donor (MAC003); CD4^+^ purified DARPin-28z-T cells induced a more rapid onset of toxicity versus unselected cells, whereas CD8^+^ purified DARPin-28z-T cells were non-toxic (Figure 5D). These data implicate CD4^+^ T cells as the main contributors to DARPin-28z-T cell toxicity and, thus, a critical factor behind our observed donor-to-donor differences, given variations in the CD4^+^:CD8^+^ T cell ratio of DARPin-28z-T cell products (Figure 4B).

Interestingly, the inter-donor disparity observed in the ratio of CD4^+^:CD8^+^ T cells in DARPin-28z-T cell products was not reflective of intrinsic differences present in PBMCs. Rather, the ratio of CD4^+^:CD8^+^ cells changed during the *ex vivo* culture period in a donor-specific manner. Unlike other donors, DARPin-28z-T cells generated from MAC026 PBMCs demonstrated an increase in their CD4^+^:CD8^+^ ratio over time (Figure S9A). Expansion data for DARPin-28z-T cell cultures generated from purified CD4^+^ or CD8^+^ T cells revealed that, while both MAC026 and MAC014 showed a similar proliferative capacity in their CD4^+^ T cells, CD8^+^ T cells from MAC026 had a diminished proliferative capacity (Figure S9B).

### Additional DARPin-28z-T Cell-Intrinsic Variables Contributed to Donor-Specific Differences in Toxicity

We postulated that, if the CD4^+^:CD8^+^ T cell ratio of the adoptive transfer product was the sole driver of donor-specific variation in our toxicity model, normalizing the dose of CD4^+^ DARPin-28z-T cells should eliminate this variation. Purified CD4^+^ DARPin-28z-T cells were generated from a panel of five different PBMC donors and delivered to tumor-bearing NRG mice at equal doses.

**Figure 6:**
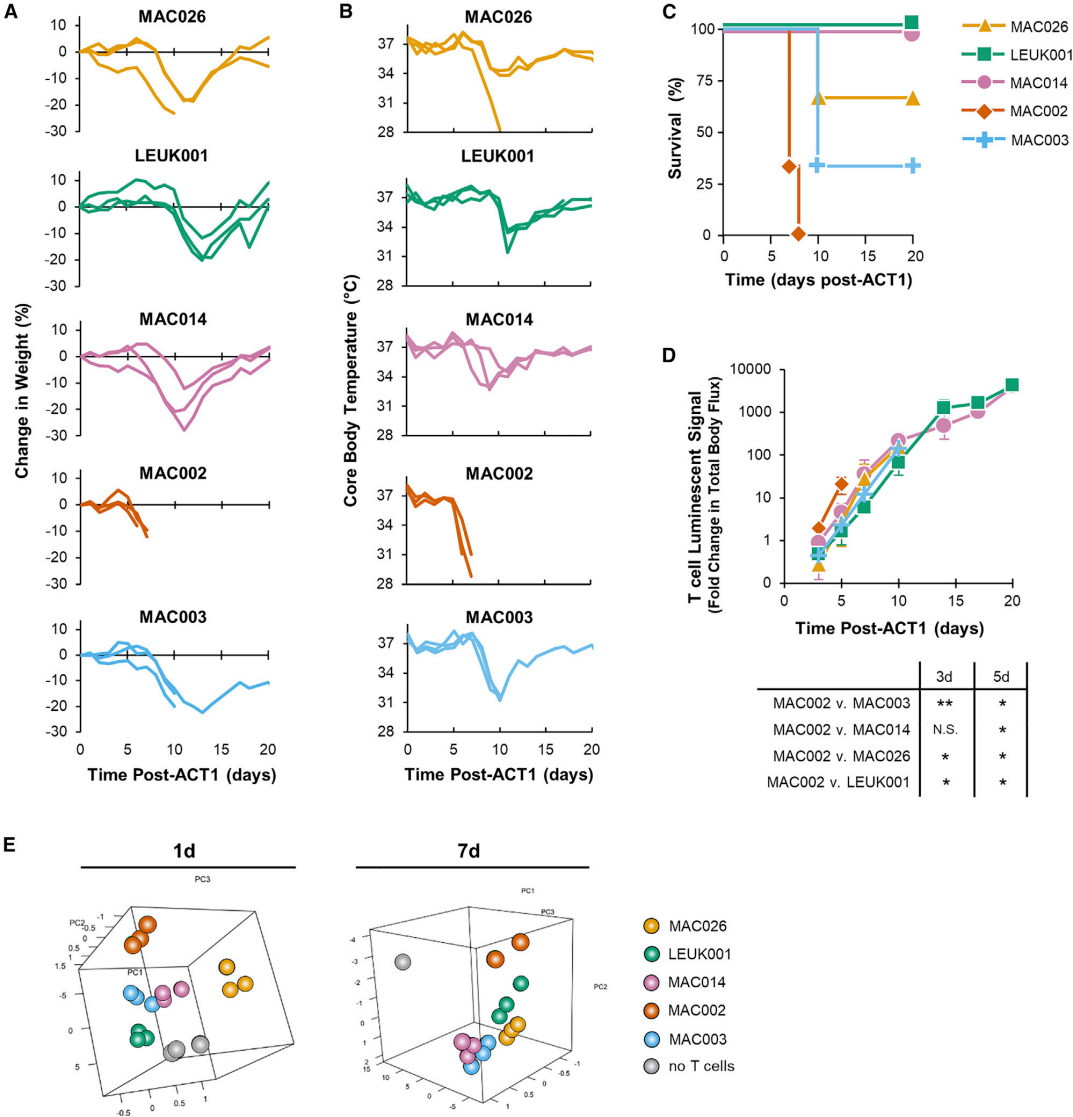
Donor-to-Donor Differences in CD4^+^ DARPin-28z-T Cell Toxicity Were Associated with Differences in Expansion and Cytokine Production Purified CD4^+^ DARPin-28z-CAR-T cells were generated from a panel of five different PBMC donors: MAC026 (gold/triangles), LEUK001 (teal/squares), MAC014 (pink/circles), MAC002 (orange/diamonds), and MAC003 (blue/crosses); cells were co-transduced with a *firefly* luciferase-expressing lentivirus. Tumor-bearing NRG mice were treated with 2.0 × 10^6^ DARPin-28z-T cells. (A–C) Mice were followed for changes in weight (A), core body temperature (B) (each line shows data from one mouse; n = 3 per donor), and survival (C). (D) Bioluminescent imaging was used to follow T cell persistence and expansion; fold change in total body flux (p/s), relative to total body flux at 1 day post-ACT1, is presented. Per donor, data indicate average values ± SD; curves end, indicating when the first mouse in group succumbed to toxicity. Unless otherwise stated, differences between donors are not significant. (E) Mice were bled at 1 and 7 days post-ACT1. Serum levels for a 13-plex panel of human cytokines were determined by multiplex analysis. Log2-transformed fluorescence intensity values were analyzed via PCA. Each sphere indicates data from one mouse. Gray spheres indicate data from no T cell control mice (vehicle treatment only). The same dataset has been analyzed by hierarchical clustering in Figure S13 and for strength of correlation with toxicity in Figure S14.

While doses of 6.0 × 10^6^ CD4^+^ DARPin-28z-T cells resulted in very similar toxicities, regardless of donor (Figure S10), donor-specific differences in the toxicity of CD4^+^ T cells were clearly resolved at the 2.0 × 10^6^ CAR-T cell dose level (Figures 6A–6C). MAC002-derived CD4^+^ DARPin-28z-T cells induced the most rapid toxicity and were uniformly lethal within 8 days of treatment. MAC026-, MAC014-, and MAC003-generated DARPin-28z-T cells all induced similar onsets in toxicity (mice experienced weight loss by 10 days post-ACT1; the average percent change in weights were −16.3% ± 5.8%, −16.2% ± 9.3%, and −16.0% ± 3.6%, respectively, at that point in time). However, MAC014-treated mice showed better overall survival. In contrast, LEUK001-derived CD4^+^ DARPin-28z-T cells showed a delay in toxicity onset (average percent change in weight, 1.0% ± 4.9% at 10 days post-ACT1, reaching −16.9% ± 4.6% at 13 days post-ACT1).

To determine whether the expansion or survival of CD4^+^ DARPin-28z-T cells could explain these donor differences, CD4^+^ DARPin-28z-T cells derived from the same five donors were co-transduced with *firefly* luciferase to permit bioluminescent imaging of CAR-T cells *in vivo* (Figure S11). At early time points, MAC002-derived CD4^+^ DARPin-28z-T cells displayed the greatest expansion when compared to CAR-T cells derived from other donors (Figure 6D), which likely contributed to their more rapid induction of toxicities. No other significant differences were observed in the *in vivo* CAR-T cell expansion between donors at any time point tested, suggesting that MAC026-, LEUK001-, MAC014-, and MAC003-derived CD4^+^ DAPRin-28z T cells all had similar expansion and survival *in vivo*.

Revisiting our earlier findings that anti-HER2 DARPin-targeted CAR scaffolds displayed a hierarchy of toxicity (28z > BBz > z), it is also worth noting that we observed a correlation between increased toxicity and expansion in this setting as well; DARPin-28z-T cells expanded to a greater extent than DARPin-BBz-T cells (Figure S12).

We next asked whether there were inter-donor differences in the intensity or patterning of cytokine release in the ensuing cytokine storm. Mice were bled 1 or 7 days post-ACT, and multiplex analysis was used to quantify the serum levels of 13 different human cytokines. Principal-component analysis (PCA) of the serum cytokine data showed tight, donor-dependent clustering, indicating that differences in the serum cytokine levels were donor dependent (Figure 6E). Hierarchical clustering of the serum cytokine data also supports donor dependency; interestingly, at each time point tested, mice treated with CD4^+^ DARPin-28z-T cells from the most rapidly toxic donor, MAC002, experienced the most severe cytokine storms, and those from the delayed toxicity donor, LEUK001, experienced the lowest levels (Figure S13). Serum levels of four different human cytokines (IFN-γ, granulocyte-macrophage colony-stimulating factor [GM-CSF], interleukin [IL]-6, and monocyte chemoattractant protein-1 [MCP-1]), across all CAR-T cell doses and donors, showed strong positive correlations between concentration in the serum and severity of toxicity as determined by Pearson’s coefficient of correlation (Figure S14), consistent with the cytokine patterns associated with CAR-T cell toxicity in humans. Acute cytokine release by the same 5-donor panel of CD4^+^ DARPin-28z-T cells, as measured by an *in vitro* co-culture assay (and, thus, independent of differences in *in vivo* expansion), confirmed that LEUK001 DARPin-28z-T cells trended toward lower levels of cytokine release (Figure S15).

Other lines of investigation failed to demonstrate correlation with the severity of toxicity in this donor panel. For example, DARPin-28z-T cells from all five donors displayed similar levels of activation and upregulation of exhaustion markers in response to HER2 stimulation (Figure S16).

In summation, these data suggest that additional CAR-T cell-intrinsic variables, beyond simply the frequency of CD4^+^ T cells in the adoptive transfer product, correlate with donor-dependent differences in the severity of DARPin-28z-T cell toxicity; in particular, a capacity for rapid expansion and an increased magnitude of cytokine release.

## Discussion

While great strides have been made toward uncovering the pathogenesis of the cytokine-associated toxicities CRS^23^^,^^24^ and ICANS,^25^^,^^26^ which have been widely observed in clinical trials of anti-CD19 CAR-T cells for hematologic malignancies, less is known about the pathogenesis of toxicities in other settings. Clinical reports of off-tumor toxicity arising from solid-tumor-targeting CAR-T cells have identified the targeted healthy tissues, but they have not addressed the fundamental features of the CAR-T cell product responsible for driving toxicity.^18^, ^19^, ^20^

Although no pre-clinical model fully recapitulates the clinical scenario, we believe that murine xenograft models of CAR-T cell toxicity afford a unique opportunity to study how the human CAR-T cell product itself contributes to toxicity in a uniform host environment. This elucidation of how CAR-T cell-intrinsic properties contribute to toxicity is occluded in clinical data by patient-to-patient variability and heterogeneity of the tumor/host microenvironment (pre-treatment regimens, tumor burden, etc.). As the development of CAR-T cells for treatment of solid tumors continues to progress, we anticipate that the observation of off-tumor toxicities in these settings will increase; a better understanding of the role played by CAR-T cell-intrinsic variables will facilitate the development of safer next-generation therapeutics.

We have described a xenograft model of off-tumor CAR-T cell toxicity where CAR-T cells targeted against human HER2 induced severe, potentially lethal, toxicities in mice. As with any model evaluating human T cell products in a murine host, the contribution of xenogeneic graft-versus-host disease must be considered. The acute nature of the observed toxicity, paired with an absence of any pathology associated with matched dosing of NGFR-T cells, supports that toxicity is a direct result of T cell activation through the anti-HER2 DARPin-targeted CAR and not the endogenous human TCR. Consistent with clinical data, we found that second-generation CAR scaffolds bearing either CD28 or 4-1BB co-stimulatory domains could produce severe toxicities. The CD28-based CAR-T cell products were more potent against solid tumors, which correlated with greater toxicity on a per-cell basis (Figure 2). Other murine models have also shown CD28-co-stimulated CAR-T cells to have increased efficacy over their 4-1BB counterparts.^27^^,^^28^ Interestingly, in one of these models, differences were also found to be more apparent at lower doses,^28^ akin to our data.

DARPin-28z-T cell-driven toxicities arose as a result of off-tumor, off-target CAR-T cell activation in pulmonary and cardiac tissues, inducing a systemic cytokine storm. While it is challenging to compare cytokine profiles between xenograft models and human clinical data (not all human cytokines are cross-reactive against their murine receptors), mice treated with DARPin-28z-T cells did display high serum levels of several cytokines that have been linked with CAR-T cell activation and toxicity in humans, including IFN-γ, GM-CSF, TNF-α, IL-2, and IL-10 (Figure 3; Table S1).^5^^,^^7^^,^^14^^,^^18^^,^^29^

Our model indicated CD4^+^ CAR-T cells as the primary contributors to DARPin-28z-T cell toxicity (Figure 5). This finding supports clinical evidence observed in a trial of carbonic anhydrase IX (CAIX)-targeted CAR-T cells where off-tumor CAR-T cell activation against target antigen expressed on bile duct epithelial cells drove a CD4^+^ T cell-biased hepatic toxicity (only scattered CD8^+^ T cells were present).^20^ At least one other pre-clinical model of CAR-T cell treatment of a solid tumor also concluded that CD4^+^ CAR-T cells played a key role in observed toxicities.^30^ Ultimately, the role of CD4^+^ T cells as a key player in the pathogenesis of CAR-T cell-associated toxicities in solid tissues will be revealed in time, as more clinical data emerge.

Our attention was drawn toward the CD4^+^ CAR-T cell population when donor differences in the severity of toxicity observed with bulk PBMC-derived (non-purified) DARPin-28z-T cell products correlated with the frequency of CD4^+^ T cells therein (Figure 4). Clinical CAR-T cell products are often manufactured from PBMCs or bulk isolated T cells, resulting in CAR-T cell products with varying CD4^+^ and CD8^+^ T cell compositions (see Table S1 in Davila et al.,^29^ or Table 1 in Kochenderfer et al.,^17^ for example). Our results provide evidence that, as suggested by others,^31^ patient-to-patient differences in the CD4^+^:CD8^+^ ratio of a CAR-T cell product contribute to differences in toxicity and support the use of defined composition autologous CAR-T cell products.^5^^,^^6^^,^^32^

Curiously, the relative toxicity of purified CD4^+^ DARPin-28z-T cell products differed between donors (Figure 6), indicating that variables beyond the CD4^+^:CD8^+^ T cell ratio contributed toward the toxic pathology. In our donor panel, MAC002-derived CD4^+^ DARPin-28z-T cells demonstrated the most rapid CAR-T cell expansion and most severe toxicity. Correlative data from clinical trials have also pointed to a relationship between CAR-T cell expansion and increased toxicity,^33^, ^34^, ^35^ although this is typically in reference to peak expansion rather than rate of expansion. Furthermore, we observed cytokine profiles that were donor specific. The cytokine profile was independent of expansion rate, as CD4^+^ DARPin-28z-T cells from all donors, except for MAC002, expanded equally. The magnitude of serum cytokines correlated with the degree of toxicity, supporting that toxicity was directly related to the magnitude of T cell activation. We suspect that the differences in cytokine profile reflect the complexity of CD4^+^ T cell differentiation. Indeed, a broad collection of CD4^+^ T cell subtypes have been identified (Th1, Th2, Th17, etc.), and the composition of these distinct CD4^+^ T cells in a CAR-T cell product will undoubtedly differ between donors.

CAR-T cells are living, cellular drugs. As such, it is conceptually intuitive that underlying differences in the cell biology of the T cell source, whether genetically encoded or environmentally established (e.g., epigenetic changes), would impact on CAR-T cell behavior. However, this has yet to be deeply explored experimentally; unlike previous pre-clinical xenograft models of CAR-T cell toxicity,^23^^,^^24^^,^^36^, ^37^, ^38^ ours specifically addresses the influence of the T cell source on toxicity. While our study revealed intriguing aspects of CAR-T cell toxicity, a thorough study of the relationship between donor background and CAR-T cell efficacy/toxicity will require a much larger donor pool; multiple models; and, ideally, patient samples where clinical toxicity outcomes are known to validate the relevance of the findings. We anticipate that a better understanding of how underlying T cell biology impacts on CAR-T cell function will inform ways to generate improved autologous CAR-T cell products or aid in donor selection for allogeneic CAR-T cell products. Investigations are ongoing.

## Materials and Methods

### Cell Lines

Human tumor cell lines OVCAR-3 and LOX-IMVI, originating from the NCI-60 panel (a kind gift from Dr. Karen Mossman, McMaster University, Hamilton, ON, Canada), were cultured in RPMI 1640 (GIBCO; Thermo Fisher Scientific) supplemented with 10% heat-inactivated fetal bovine serum (FBS; GIBCO), 2 mM L-glutamine (BioShop, Burlington, ON, Canada), 10 mM HEPES (Roche Diagnostics, Laval, QC, Canada), 100 U/mL penicillin + 100 μg/mL streptomycin (GIBCO), and 55 μM β-mercaptoethanol (GIBCO). Prior to their use, parental OVCAR-3 cells were subjected to an *in vivo* passage. In short, OVCAR-3 cells were injected subcutaneously (s.c.) into the hindflank of an NRG mouse and allowed to grow for 72 days prior to harvest, digestion (incubation with a mixture of collagenase type I [GIBCO], DNase I [Roche], and hyaluronidase [MP Biomedicals, Solon, OH, USA]), and *ex vivo* expansion. All cell lines were grown at 5% CO_2_, 95% air, and 37°C. All cell lines tested negative for mycoplasma contamination (LookOut Mycoplasma PCR Detection Kit, Sigma-Aldrich Canada, Oakville, ON, Canada). Expression status of HER2 on the surface of these cell lines was verified by flow cytometry; cells were stained with Herceptin (anti-hHER2; a kind gift from Dr. Ronan Foley, Juravinski Hospital and Cancer Centre, Hamilton, ON, Canada) followed by phycoerythrin (PE)-conjugated goat anti-human immunoglobulin G (IgG) secondary antibody (catalog no. 109-115-098, Jackson ImmunoResearch, West Grove, PA, USA). Cells were used within 2 weeks of thaw for *in vivo* inoculation and *in vitro* assays.

### Generation of CAR Lentiviral Vectors

Generation of the DARPin-28z-CAR (consisting of the IgGκ leader, anti-HER2 H10-2-G3 DARPin, human myc tag, BamHI site, CD8α hinge, CD28 TM and cytoplasmic domains, CD3ζ cytoplasmic tail, and NheI site) was previously described.^22^ The DARPin-BBz-CAR (consisting of the IgGκ leader, anti-HER2 H10-2-G3 DARPin, human myc tag, BamHI site, CD8α hinge and TM, 4-1BB cytoplasmic domain, CD3ζ cytoplasmic tail, and NheI site) was generated by cloning the CD8α hinge and TM, 4-1BB cytoplasmic domain, and CD3ζ cytoplasmic tail portions from an anti-CD19 CAR (prepared according to Brogdon et al.^39^) between the BamHI and NheI sites of the DARPin-28z-CAR. To generate the DARPin-z-CAR, overlap extension PCR was used to delete the 4-1BB sequence from the DAPRin-BBz CAR. To facilitate the production of third-generation lentiviruses, the transfer plasmid pCCL was used (a kind gift from Dr. Megan Levings, University of British Columbia, Vancouver, BC, Canada).^40^ The parental pCCL vector consists of a bi-directional promoter system; tNGFR (truncated NGFR (CD271); used as a transduction control) is expressed under control of the minimal cytomegalovirus promoter (mCMV), and the human elongation factor 1 alpha (EF-1α) promoter lacks a transgene (the parental pCCL vector was used to generate receptor negative control T cells). CARs were cloned into pCCL under the control of the EF-1α promoter. Luciferase expression was achieved using a variant of the pCCL plasmid in which puromycin resistance was encoded under the mCMV promoter and an enhanced firefly luciferase^41^ was encoded under the EF-1α promoter.

### Lentivirus Production

Self-inactivating, non-replicative lentivirus was produced using a third-generation system, which has been previously discussed.^42^^,^^43^ Briefly, 8 × 10^6^ HEK293T cells cultured on 15-cm-diameter tissue-culture-treated dishes (NUNC; Thermo Fisher Scientific) were transfected with the packaging plasmids pRSV-Rev (6.25 μg), pMD2.G (9 μg), pMDLg-pRRE (12.5 μg), and the desired pCCL transfer plasmid (described earlier; 32 μg) using Opti-MEM (GIBCO; Thermo Fisher Scientific) and Lipofectamine 2000 (Thermo Fisher Scientific). Twelve to 16 h after transfection, media were replaced; fresh medium was supplemented with sodium butyrate (1 mM; Sigma-Aldrich). Cell-culture supernatant, containing lentiviral particles, was collected after 36–48 h, and lentivirus was isolated by ultracentrifugation. Lentiviruses were stored at −80°C. Viral titer in transduction units (TU)/mL was determined by serial dilution and transduction of HEK293T cells with virus (transduction after ~72 h was measured as percent tNGFR^+^ via flow cytometry using an anti-NGFR-VioBrightFITC antibody [ME20.4-1.H7, catalog no. 130-104-847, Miltenyi Biotec, Bergisch Gladbach, Germany]).

### Transduction of Human T Cells

This research was approved by the Hamilton Integrated Research Ethics Board, which operates in compliance with the International Council for Harmonisation of Technical Requirements for Pharmaceuticals for Human Use (ICH) Good Clinical Practice Guidelines, the Tri-Council Policy Statement: Ethical Conduct for Research Involving Humans, Division 5 Health Canada Food and Drug Regulations, and the Declaration of Helsinki. All PBMC donors in this study provided informed written consent. Lentivirus-engineered human T cells were generated as previously described.^43^ Human PBMCs from healthy donors (McMaster Immunology Research Centre [MIRC] adult cohort; MAC) or commercial leukapheresis products (LEUK; HemaCare, Van Nuys, CA, USA) were isolated by Ficoll-Paque-Plus gradient centrifugation (GE Healthcare, Baie d’Urfe, QC, Canada) and cryopreserved in inactivated human AB serum (Corning, Corning, NY, USA) containing 10% DMSO (Sigma-Aldrich Canada). T cells were activated from PBMCs with anti-CD3/28 Dynabeads at a 0.8:1 bead-to-cell ratio (GIBCO) following manufacturer’s guidelines and were cultured in T cell media (RPMI 1640 [GIBCO] supplemented with 10% heat-inactivated FBS [GIBCO], 2 mM L-glutamine, 10 mM HEPES, 1 mM sodium pyruvate [Sigma-Aldrich Canada], 1× non-essential amino acids [GIBCO], 55 μM β-mercaptoethanol, 100 U/mL penicillin + 100 μg/mL streptomycin, 660 IU rhL-2. and 10 ng/mL rhIL-7 [PeproTech, Rocky Hill, NJ, USA]). After 18–24 h, cells were transduced with lentivirus at a multiplicity of infection (MOI) of 2–5. In cases of co-transduction for luciferase expression, a second lentivirus was added 6–12 h later at an MOI of 2. Cells were monitored daily and fed T cell media according to cell counts every 2–3 days to maintain a concentration of 1 × 10^6^ cells per milliliter for a period of 11–14 days prior to use *in vitro* and/or *in vivo*. Purified CD4^+^ or CD8^+^ T cells were generated using the same protocol, except that CD4^+^ or CD8^+^ T cells were isolated from PBMCs, prior to activation, using magnetic negative selection (catalog no. 19052 and catalog no. 19053, STEMCELL Technologies, Vancouver, BC, Canada), according to the manufacturer’s instructions.

### Phenotypic Analysis by Flow Cytometry

Cell surface phenotyping of CAR- or control-T cells was evaluated by direct staining with Alexa Fluor 700-conjugated anti-CD4 (clone: OKT4, catalog no. 56-0048-82, eBioscience and Thermo Fisher Scientific), PerCP-Cyanine5.5-conjugated anti-CD8 (clone: RPA-T8, catalog no. 45-0088-42, eBioscience), and BV421-conjugated anti-tNGFR (clone: C40-1457, catalog no. 562562, BD Biosciences). Detection of CAR expression was determined in a two-step stain by indirect immunofluorescence; incubation with rhHER2-Fc chimeric protein (catalog no. 1129-ER-050, R&D Systems, Minneapolis, MN, USA) was followed by a PE-conjugated goat anti-human IgG secondary antibody (catalog no. 109-115-098, Jackson ImmunoResearch). Detection of cytosolic luciferase was determined via intracellular cytokine staining (ICS); in brief, cells were fixed and permeabilized according to the BD Cytofix/Cytoperm Fixation and Permeabilization Kit (catalog no. 554714, BD Biosciences), and luciferase expression was determined in a two-step stain by indirect immunofluorescence (incubation with anti-Luc [clone: Luci17, catalog no. ab16466, Abcam] was followed by a PE-conjugated goat anti-mouse IgG secondary antibody [catalog no. 115-116-146, Jackson ImmunoResearch]). All stains were conducted at room temperature for 30 min unless otherwise stated. All flow cytometry was conducted on a BD LSRFortessa or BD LSRII cytometer (BD Biosciences) and analyzed using FlowJo vX software (FlowJo, Ashland, OR, USA).

### Functional Analysis of CAR-T Cells following Stimulation with Tumor Cell Lines

5 × 10^5^ CAR-T cells were stimulated with 5 × 10^4^ HER2^+^ (OVCAR-3) or HER2^−^ (LOX-IMVI) tumor cells for 4 h at 37°C in a round-bottomed 96-well plate. Brefeldin A (BD GolgiPlug Protein Transport Inhibitor; catalog no. 555029, BD Biosciences) was added at the start of stimulation following the manufacturer’s instructions. After stimulation, cells were stained for desired surface markers as described earlier. BD Cytofix/Cytoperm (as described earlier) was used to permit ICS, and cells were stained directly for fluorescein isothiocyanate (FITC)-conjugated anti-TNF-α (clone: MAb11, catalog no. 554512, BD Biosciences), and allophycocyanin (APC)-conjugated anti-IFN-γ (clone: B27, catalog no. 554702, BD Biosciences) expression. Flow cytometry and data analysis was conducted as described earlier.

### *In Vitro* Cytotoxicity Assay

Adherent tumor cell lines were plated at 1.25 × 10^4^ cells per well (OVCAR-3) or 2.5 × 10^4^ cells per well (LOX-IMVI) in a 96-well flat-bottomed tissue-culture-treated plate and allowed to rest overnight. CAR-T cell cultures (a mix of NGFR^+^ and non-transduced T cells) were added at various effector:target (E:T) ratios (from 0.25:1 to 8:1) in triplicate, and co-cultures were incubated for 6 h at 37°C. To resolve cytotoxicity, wells were washed 3× with warmed PBS to remove any non-adherent cells, and 100 μL 10% solution of alamarBlue Cell Viability Reagent (Life Technologies) in T cell media was added. After a 3- to 4-h incubation at 37°C, color change was measured by fluorescence (excitation, 530 nm; emission, 595 nm) on a Synergy plate reader (BioTek, Winooski, VT, USA). Tumor cell viability was calculated as the loss of fluorescence in experimental wells compared to untreated target cells.

### Mice

All animal studies were approved by the McMaster University Animal Research Ethics Board. 5-week-old female NOD.Cg-Rag1^tm1Mom^Il2rg^tm1Wjl^/SzJ (NRG) mice were purchased from The Jackson Laboratory (stock no. 007799, Bar Harbor, ME, USA), or bred in house.

### Adoptive Transfer and *In Vivo* Monitoring

Mice (6–12 weeks old) were implanted with 2.5 × 10^6^ OVCAR-3 cells s.c. on the right hindflank. After 35–56 days of tumor growth, mice were optimized into treatment groups based on tumor volume;^44^ average tumor volume at the time of treatment was 155 mm^3^. CAR-T cells were infused intravenously (i.v.) (deemed as ACTs) through the tail vein as two doses delivered 48 h apart in 200 μL sterile PBS (T cells were days 14 and 16 in culture on respective treatment days; doses as specified in the text and figure legends represent the total sum of effective (NGFR^+^) T cells received per mouse). Tumor volume was measured by caliper (catalog no. 500-196-30, Mitutoyo Canada, Toronto, ON, Canada) every 2–3 days post-ACT and calculated, in cubic millimeters, as length × width × height; percent change in tumor volume was calculated as: [(current volume − pre-ACT volume)/pre-ACT volume] ⋅ 100. Core body temperature (in degrees Celsius, via rectal probe; catalog no. 23609-230, VWR) and weight (in grams, via scale; catalog no. 01922406, OHAUS, Parsippany, NJ, USA) were measured every 1–3 days post-ACT; percent change in weight was calculated as: [(current weight − pre-ACT weight)/pre-ACT weight] ⋅ 100. Luciferase-engineered T cells were monitored through bioluminescent imaging every 1–9 days post-ACT1. In short, mice received an intraperitoneal injection of 150 mg/kg D-Luciferin (PerkinElmer; Waltham, MA, USA), and ventral images were collected 14 min later using an IVIS Spectrum (Caliper Life Sciences; Waltham, MA, USA). Images were analyzed using Living Image Software, v.4.2, for Mac OS X (PerkinElmer). Fold change in whole body total flux (measured in photons per second; p/s) relative to 1 day post-ACT1 was calculated as: [(current flux − flux at 1 day post-ACT1)/flux at 1 day post-ACT1]. Measurements of overall toxicity and efficacy encompassing the duration of the experiment were calculated as net area under the curve (using GraphPad Prism, v.6.01) for percent weight loss over time or percent change in tumor volume over time graphs, respectively (baseline at y = 0, peaks below baseline included).

### Lung Homogenate Stimulation

PBS-perfused lungs were excised from tumor-free NRG mice. Lung tissue was mechanically disrupted, digested in a type I collagenase (1.5 mg/mL) + DNase I (0.4 mg/mL) solution for 1 h at 37°C, and filtered (70 μm) to generate a single cell suspension. Engineered T cells were stained with CellTrace Violet (CTV; Thermo Fisher Scientific, catalog no. C34557) prior to co-culture with lung homogenates at a 1:1 ratio. After 4 days, T cell populations were evaluated by flow cytometry (gating strategy: lymphocytes → singlets → live cells → NGFR^+^ → CD4^+^ or CD8^+^ → CTV histogram).

### Serum Cytokine Analysis

Whole blood was collected via a terminal or non-terminal retro-orbital bleed. Serum was isolated using CAPIJECT capillary blood collection serum tubes according to the manufacturer’s instructions (catalog no. T-MG, Terumo Medical, Somerset, NJ, USA). Quantification of 13 human cytokines and chemokines (catalog no. HDF13) or 31 murine cytokines and chemokines (catalog no. MD31) was performed in a multiplex assay by Eve Technologies (Calgary, AB, Canada) using the Bio-Plex 200 System and MILLIPLEX assay kits from Millipore. The assay sensitivities of these markers ranged from 0.1 to 9.5 pg/mL (human) and from 0.1 to 33.3 pg/mL (murine); individual analyte values can be found on the Eve Technologies website. Prior to downstream analysis, fluorescence intensity values were transformed to the log2 scale.^45^ Heatmaps (Figure 2; Figure S5) were created using HeatMapViewer v.13.9, available on GenePattern (https://cloud.genepattern.org/gp/pages/login.jsf). After preprocessing, we confirmed that samples were separated into homogeneous groups matching experimental groups and performed PCA (princomp function from the “stats” and “rgl”^46^ packages in R) with all 13 human cytokines. Heatmaps (Figure S13) were generated using the “gplots” package^47^ in R. Linear models were fit for each cytokine using the “limma” package in R to test for differential expression for pre-specified contrasts.^48^ The p values for each contrast were obtained for each cytokine and adjusted for multiple comparisons using the Benjamini–Hochberg procedure.^49^

### Histology

Tissues were prepared for veterinary necropsy via whole-body formalin perfusion as described previously.^50^ Total body necropsy included collection of salivary gland, lung/trachea, heart, diaphragm, liver, small intestine, cecum, kidney/adrenal gland, spleen/pancreas, stomach, female genital tract, brain, and subcutaneous tumor; a repeat experiment was conducted to confirm observations focused on heart, lung, and subcutaneous tumor tissue. After fixation in 10% neutral buffered formalin, tissues were paraffin-embedded, sectioned, and stained using H&E or IHC for expression of human CD3 (Abcam, catalog no. ab16669, Toronto, ON, Canada) (conducted using the Leica BOND RX [Leica Biosystems, Concord, ON, Canada]). Aforementioned histology services were performed by the John Mayberry Histology facility at the McMaster Immunology Research Centre. Opal multiplex immunofluorescence was performed by the Molecular and Cellular Immunology Core at the British Columbia Cancer Agency’s Deeley Research Centre. In short, formalin-fixed paraffin-embedded (FFPE) tissue sections were stained with anti-CD4 (ab133616, Abcam, catalog no. EPR6855) detected with Opal 520 (PerkinElmer, NEL797001KT), anti-CD8 (SP16, Spring Biosciences, catalog no. M3162) detected with Opal 650 (PerkinElmer, NEL797001KT), anti-HER2 (polyclonal, Cell Signaling Technology, catalog no. 2242) detected with Opal 570 (PerkinElmer, NEL797001KT), anti-pan-CK (PCK-26, Sigma-Aldrich, catalog no. C1801) detected with Opal 690 (PerkinElmer, NEL797001KT), anti-Ki-67 (SP6, Spring Biosciences, catalog no. M3062) detected with Opal 620 (PerkinElmer, NEL797001KT), and DAPI (PerkinElmer, NEL797001KT). Multispectral images (20× magnification, 3 fields per tumor, and 3 fields containing perivascular sites per lung) were collected using the PerkinElmer Vectra System. Quantification was performed using inForm Advanced Image Analysis Software (PerkinElmer). Blinded pathologic assessment of H&E and CD3 IHC slides was performed by a veterinary pathologist (Dr. Jacek Kwiecein, McMaster University).

### Statistics

One-way ANOVA was used to determine whether any statistically significant differences existed in the means of three or more groups (alpha = 0.05). Student’s t tests, two-tailed, type two or three (depending on variance), were used to compare data between two groups and as a post hoc test for ANOVA results. Strength of linear correlation was determined using the Pearson correlation coefficient. Results were prepared using Microsoft Excel 2010. Log-rank tests were used to compare survival using GraphPad Prism v.6.01 for Windows (GraphPad Software, La Jolla, CA, USA). Significant differences were defined as: ∗p < 0.05, ∗∗p < 0.01, and ∗∗∗p < 0.001 (N.S. indicates not significant).

## Author Contributions

J.A.H. and J.L.B. conceived of these studies, designed experiments, and wrote the manuscript. J.A.H. acquired and analyzed all data (unless otherwise stated). J.M.K. performed pathological analysis of murine tissues. V.W.C.L. designed and performed lung homogenate stimulations. A.D.-G. performed PCA and hierarchical clustering. C.B., K.B., and C.A. assisted with *in vivo* experiments. Y.W. assisted with exhaustion experiments. C.W.H. and G.F.D. made contributions to receptor design. H.D. and K.M. performed and analyzed multiplex immunofluorescence as designed by B.H.N., K.M., and J.L.B. All authors reviewed the final manuscript prior to submission.

## Conflicts of Interest

J.A.H. is a co-inventor on a chimeric receptor patent. C.W.H. has ownership interest in Triumvira Immunologics and is a co-inventor on a chimeric receptor patent. B.H.N. is a consultant for Symvivo and Immunovaccine. J.L.B. has ownership interest in and receives research funding from Triumvira Immunologics. J.L.B. is a co-inventor on several patents related to chimeric receptors and oncolytic viruses. The other authors declare no competing interests.

## Supplemental Information

Document S1. Figures S1–S16, Tables S1 and S2, and Supplemental Materials and Methods

Document S2. Article plus Supplemental Information
