# Supplementary material for: A Cross-Reactive Small Protein Binding Domain Provides a Model to Study Off-Tumor CAR-T Cell Toxicity
Source: Mol Ther Oncolytics. 2020 Apr 14;17:278–92. doi: 10.1016/j.omto.2020.04.001 (PMC7191649; doi:10.1016/j.omto.2020.04.001)
Supplement: Document S1. Figures S1–S16, Tables S1 and S2, and Supplemental Materials and Methods [file mmc1.pdf]

OMTO, Volume *17*

Supplemental Information

A Cross-Reactive Small Protein Binding Domain Provides a Model to Study Off-Tumor CAR-T Cell Toxicity

Joanne A. Hammill, Jacek M. Kwiecien, Anna Dvorkin-Gheva, Vivian W.C. Lau, Christopher Baker, Ying Wu, Ksenia Bezverbnaya, Craig Aarts, Christopher W. Heslen, Galina F. Denisova, Heather Derocher, Katy Milne, Brad H. Nelson, and Jonathan L. Bramson

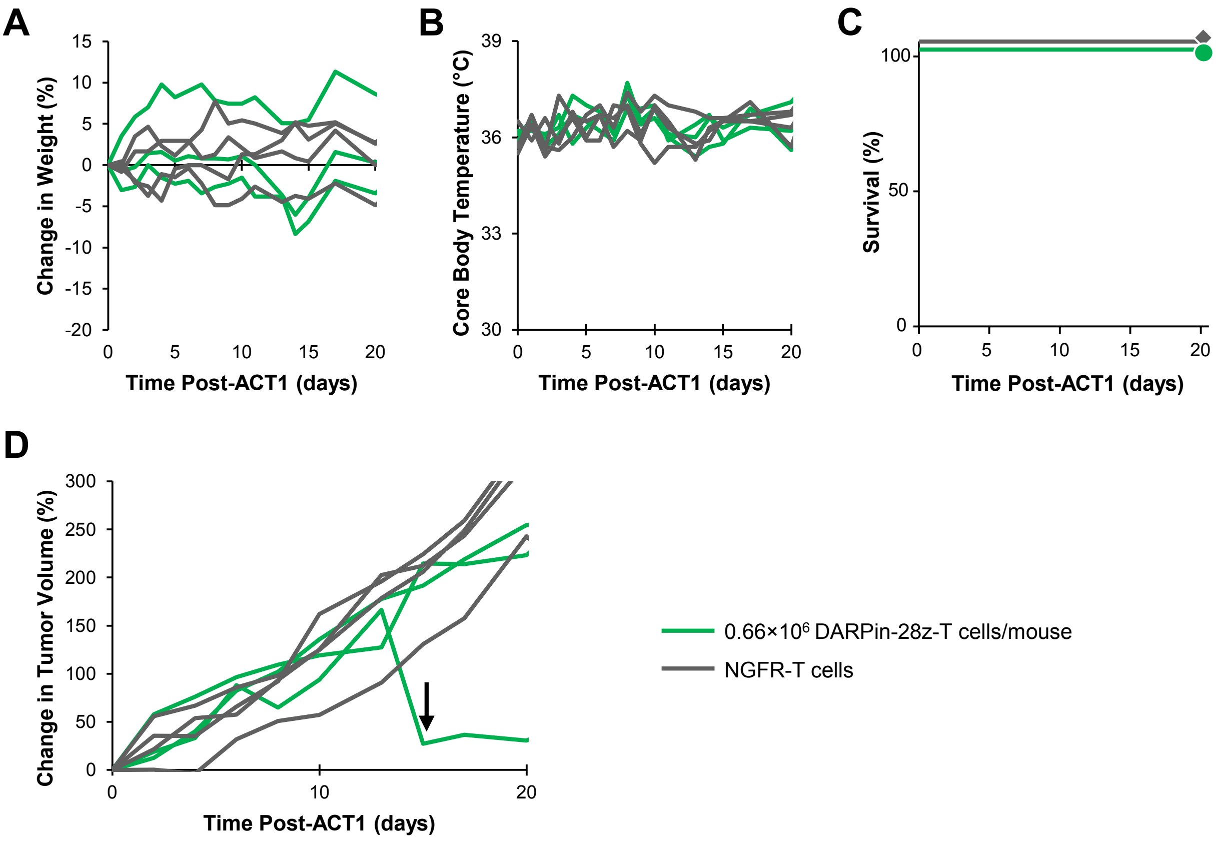

Supplemental Figure 1. Low doses of DARPin-28z-T cells were well-tolerated, but lacked anti-tumor efficacy. OVCAR-3 tumor-bearing NRG mice were treated with 0.66×10^6 DARPin-28z-T cells or a matched number or NGFR-T cells. Mice were followed for changes in weight (**A**), core body temperature (**B**), survival (**C**), and tumor volume (**D**). Each curve indicates data from one mouse. Arrow indicates a tumor ulceration event; fluid loss causes a rapid decrease in tumor volume. In our experience, these are independent of anti-tumor efficacy as the phenomenon is regularly observed in large control tumors.

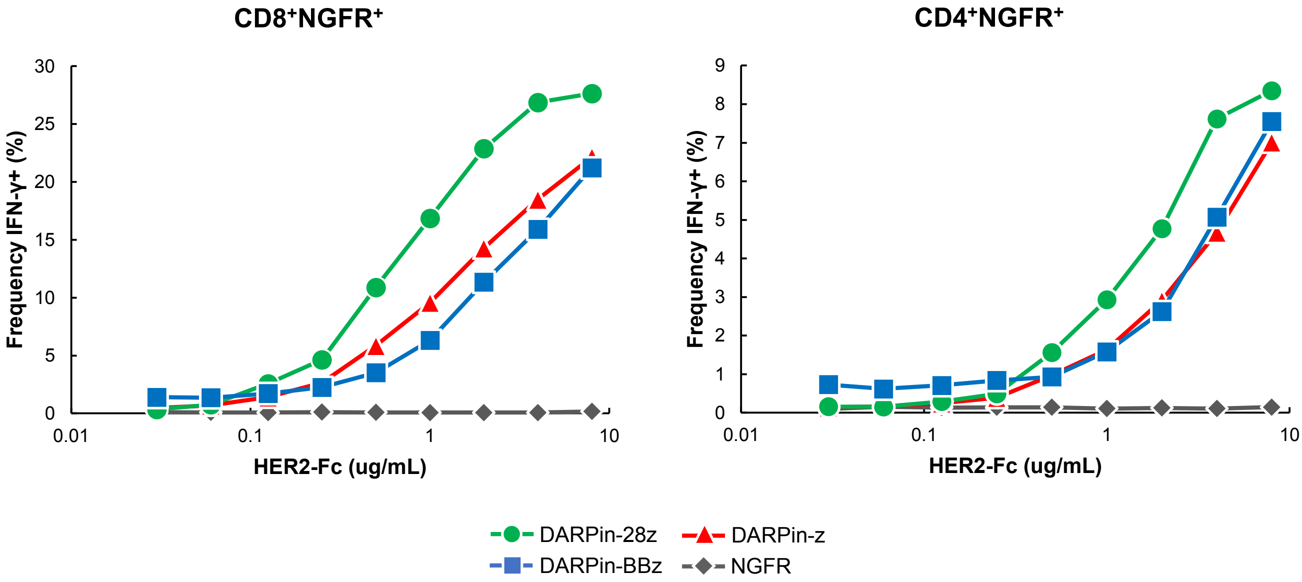

Supplemental Figure 2. Functional avidity of DARPin-28z-, BBz-, and z- CAR-T cells. A 96-well plate was coated with recombinant HER2-Fc at concentrations as indicated. CAR- or NGFR-T cells were stimulated in the plate for 4 hours prior to intracellular cytokine staining. Production of the activation cytokine IFN-γ was measured by flow cytometry (upstream gating strategy: lymphocytes → singlets → $NGFR^{+}CD4^{+}$ *or* $NGFR^{+}CD8^{+}$ T cells).

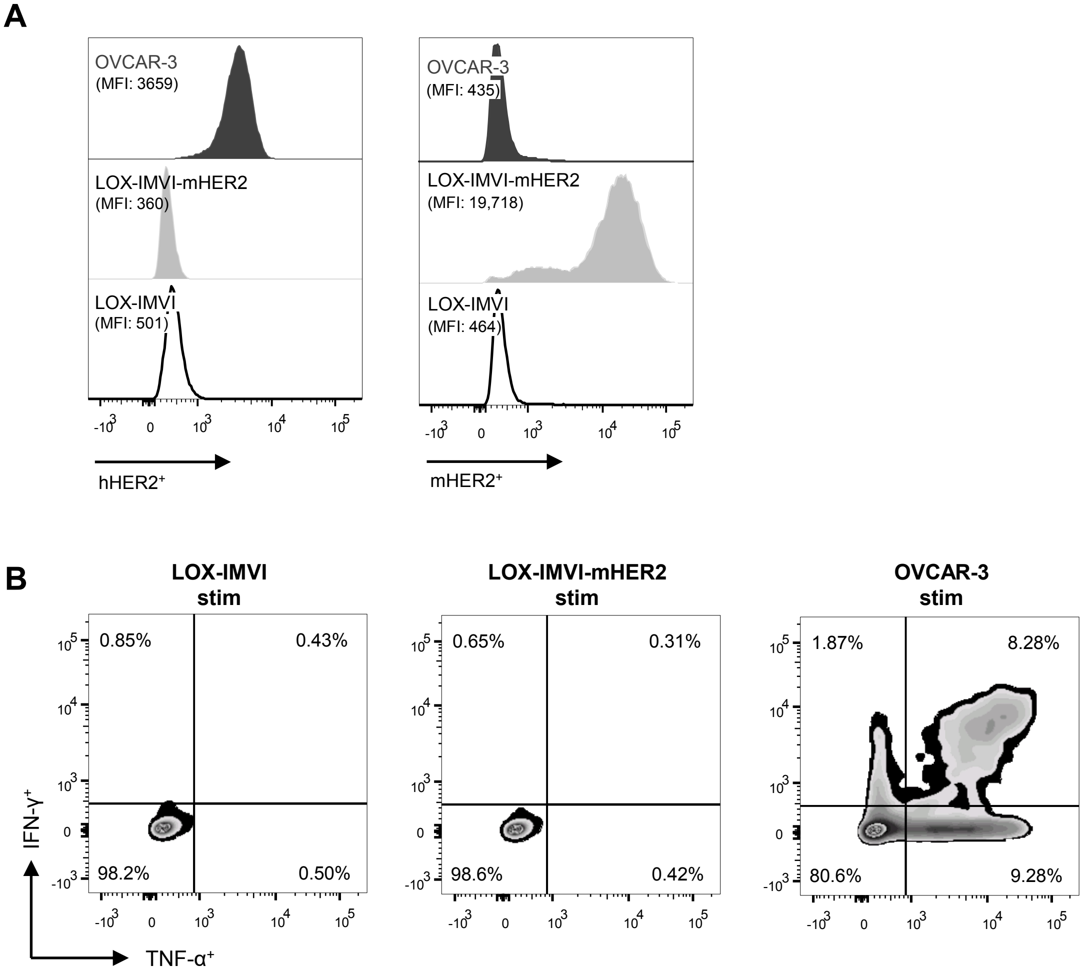

Supplemental Figure 3. DARPin-28z is not cross reactive against murine HER2. A. Expression of human or murine HER2 (hHER2 or mHER2, respectively) on OVCAR-3, LOX-IMVI, and LOX-IMVI-mHER2 tumor cell lines as determined by flow cytometry. **B.** Production of activation cytokines (IFN-γ and TNF-α) by MAC026 $CD8^+$ DARPin-28z-T-cells after stimulation with tumor cell lines (as indicated) was determined by flow cytometry. Data is representative of findings from two independent experiments.

A

CD4+	NGFR	DARPin-28z
Unstimulated	1	1
Brain	1	1
Heart	1	3.77
Kidney	1.11	1.62
Liver	1.14	1.04
Lung	1.02	5.82

CD8+	NGFR	DARPin-28z
Unstimulated	1.02	1.02
Brain	1	1.02
Heart	1	4.1
Kidney	1.16	1.88
Liver	1.03	1.09
Lung	1	5.61

B

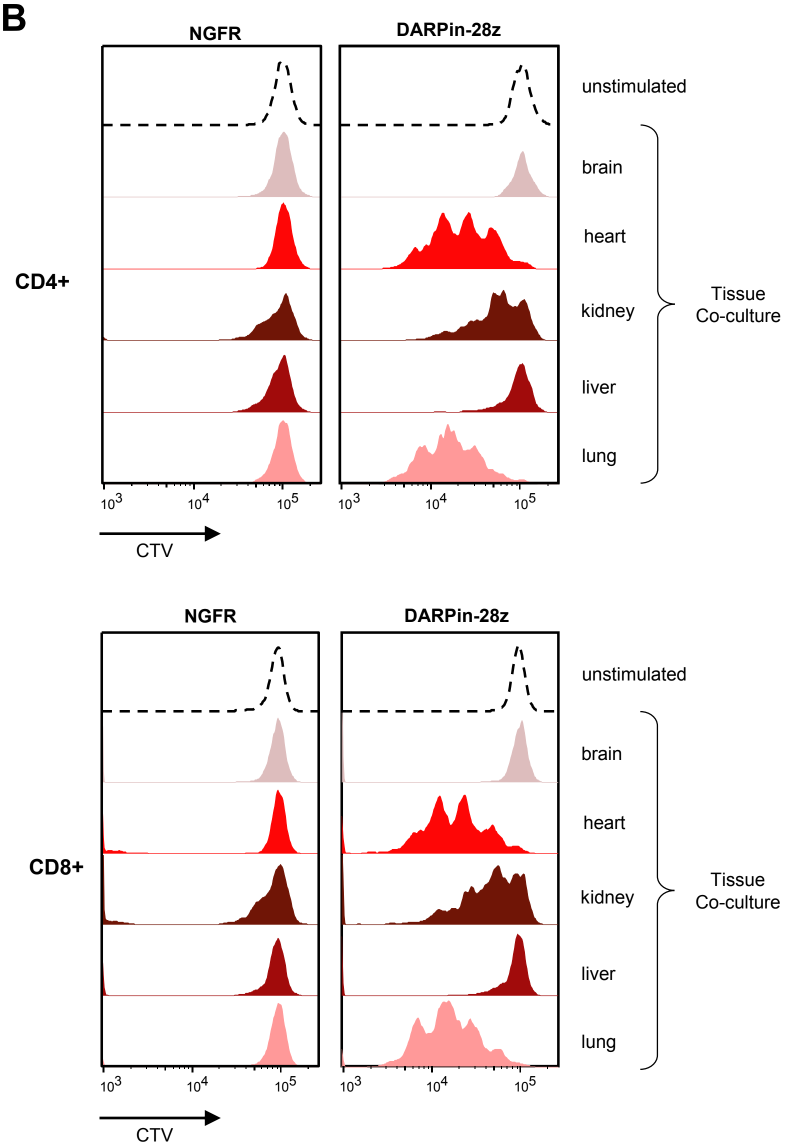

Supplemental Figure 4. DARPin-28z-T cells proliferate strongly when stimulated with murine lung or heart homogenates. DARPin-28z- or NGFR-T cells were co-cultured with tissue homogenates from tumor-free NRG mice at a 1:1 ratio for 4 days. T cell proliferation was measured by flow cytometry (upstream gating strategy: singlets → live cells → $NGFR^+$ → $CD4^+$ or $CD8^+$ T cells, as indicated) using CellTrace Violet (CTV) dye. **A.** Proliferation index values (the average number of cells that resulted per initial cell). **B.** Histogram plots.

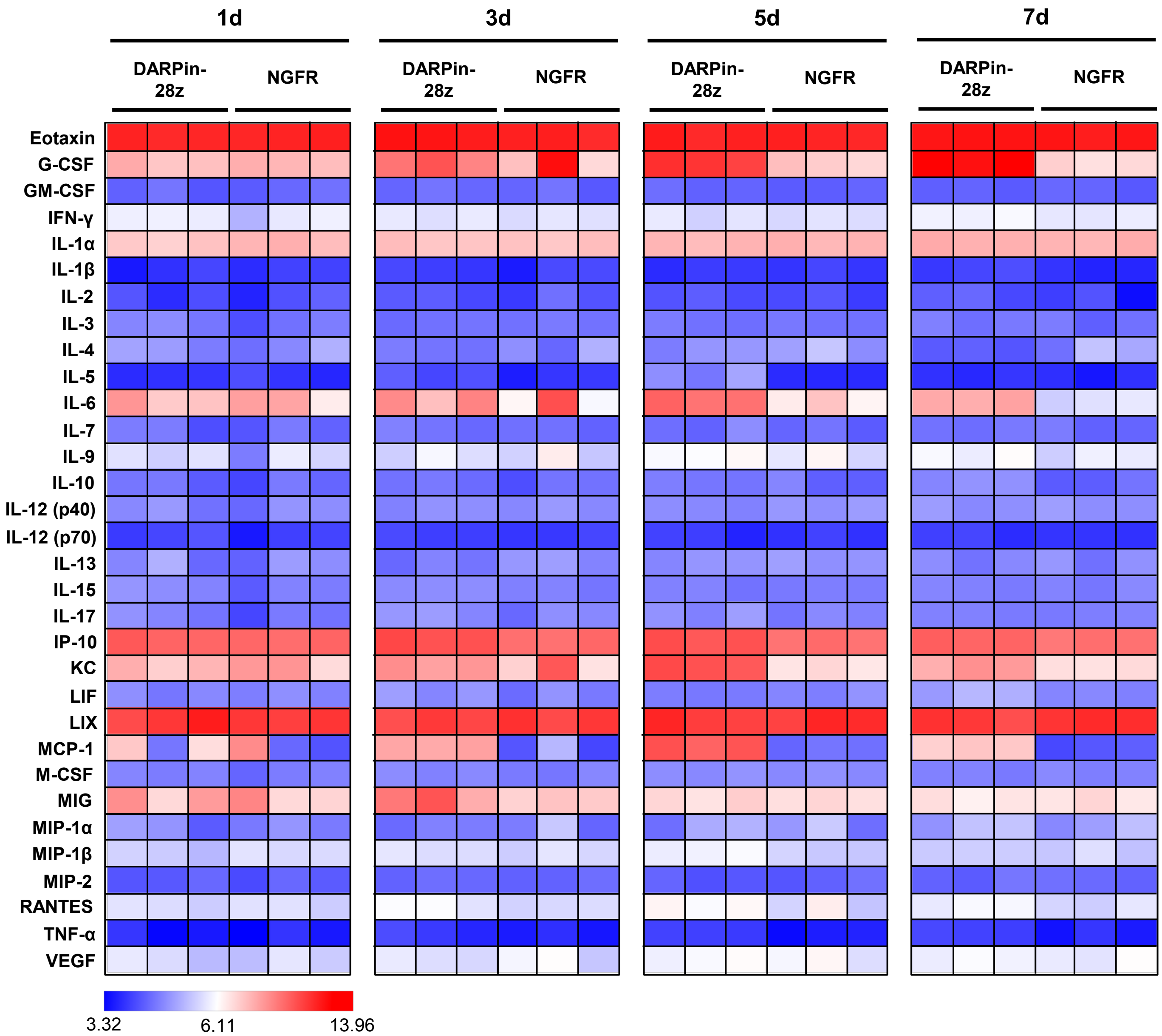

Supplemental Figure 5. Murine serum cytokine levels after DARPin-28z-T cell treatment. OVCAR-3 tumor-bearing NRG mice were treated with 6×10^6 effective DARPin-28z-T cells (or an excess number of donor-matched NGFR-T cells). Mice (n = 3) were bled at 1, 3, 5, or 7d post-ACT1 for multiplex analysis of murine serum cytokine content. A globally normalized heat map of log2-transformed fluorescence readings was generated. Each square displays data from one mouse. Colorimetric scale bar indicates minimum, average, and maximum values on map. Absolute values are displayed in **Supplemental Table 2**.

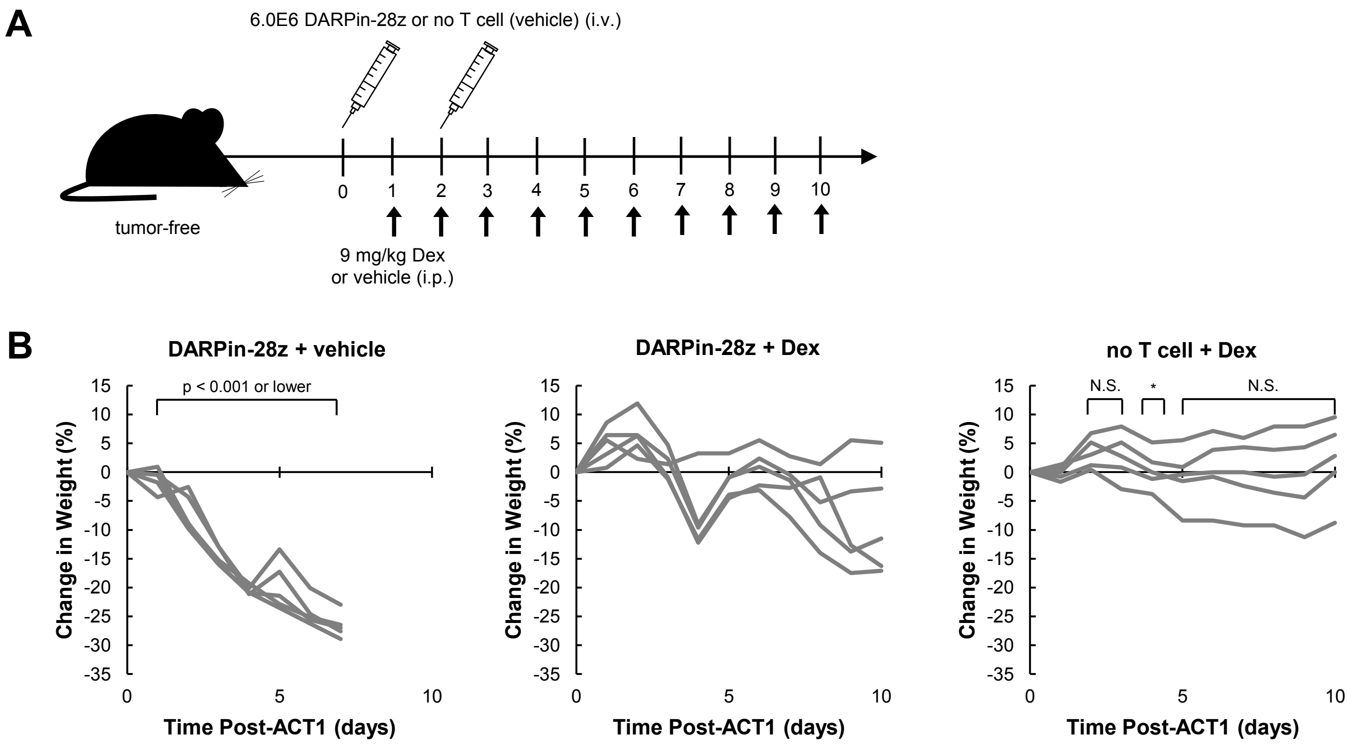

Supplemental Figure 6. DARPin-28z-T cell toxicity is mitigated by corticosteroid treatment. Tumor-free NRG mice received 6.0E6 DARPin-28z-T cells or vehicle-only control (no T cells). Starting 24 hours post-ACT1, mice received 9 mg/kg Dexamethasone (Dex) or vehicle; dosing schedule as illustrated (**A.**). Mice were monitored over time for changes in weight (**B.**). Each curve shows data from one mouse; curves end when mouse succumbed to toxicity. Statistics make comparisons to the DARPin-28z + Dex group.

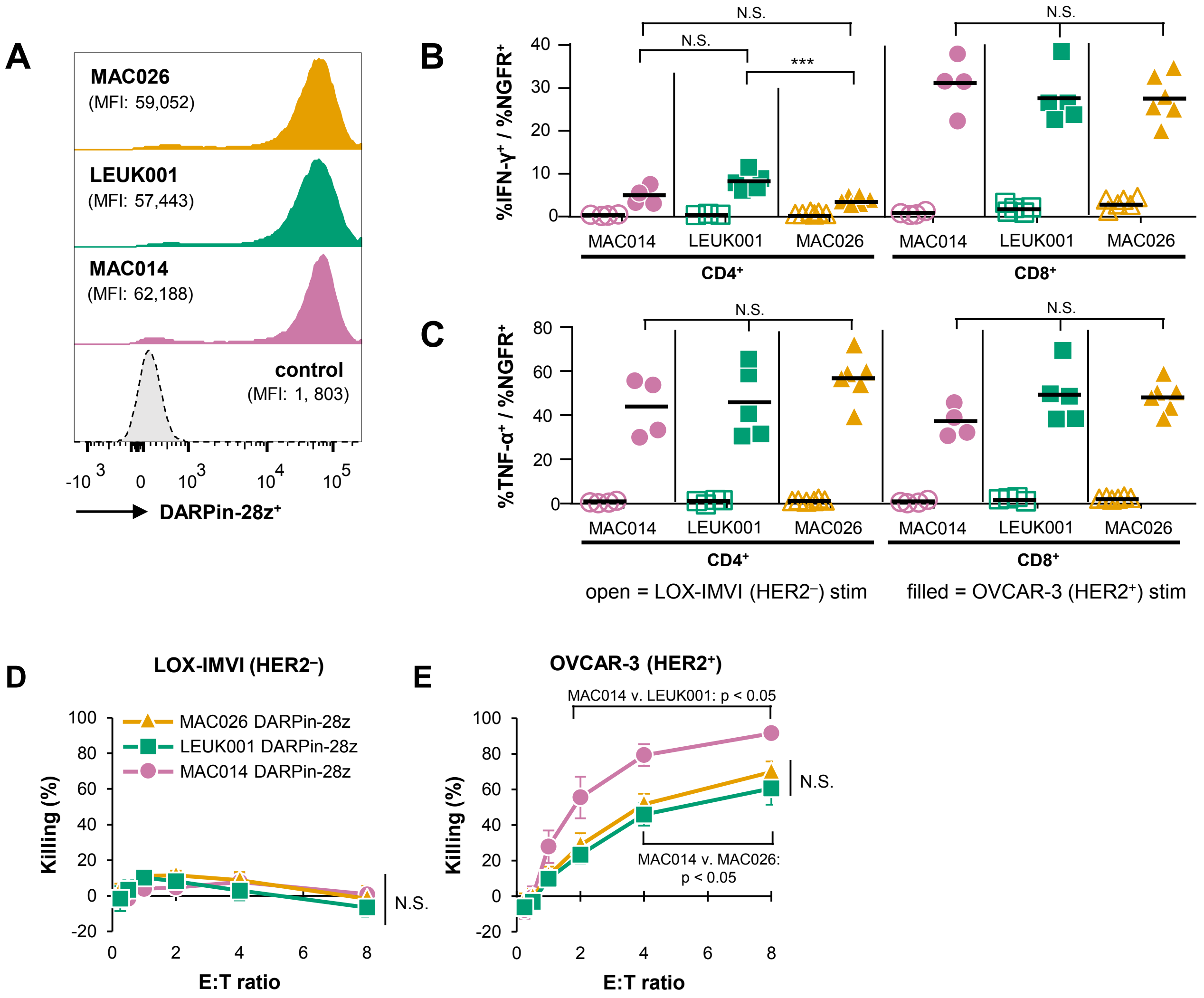

Supplemental Figure 7. *In vitro* comparison of DARPin-28z-T cells manufactured from unique PBMC sources. A. Expression of DARPin-28z on the surface of transduced T cells (upstream gating strategy: lymphocytes → singlets → NGFR+) generated from three different PBMC sources (donors: MAC026 (gold/triangles), LEUK001 (teal/squares), or MAC014 (pink/circles)) as determined by flow cytometry and compared to a secondary only staining control (dashed histogram). Results have been replicated in an additional independent experiment. **B**-**C**. Production of IFN-γ (**B**) and TNF-α (**C**) by CD4+ or CD8+ DARPin-28z-T cells after exposure to HER2+ (OVCAR-3; filled symbols) or HER2– (LOX-IMVI; open symbols) tumor cell lines. Each data point shows data from a single independent experiment (n = 4-6 per donor); black lines indicate mean values. **D**-**E**. Cytotoxicity against HER2– (**D**) or HER2+ (**E**) tumor cell lines. Error bars = SEM. Data from n = *x* independent experiments; MAC014 = 4, LEUK001 = 5, MAC026 = 6.

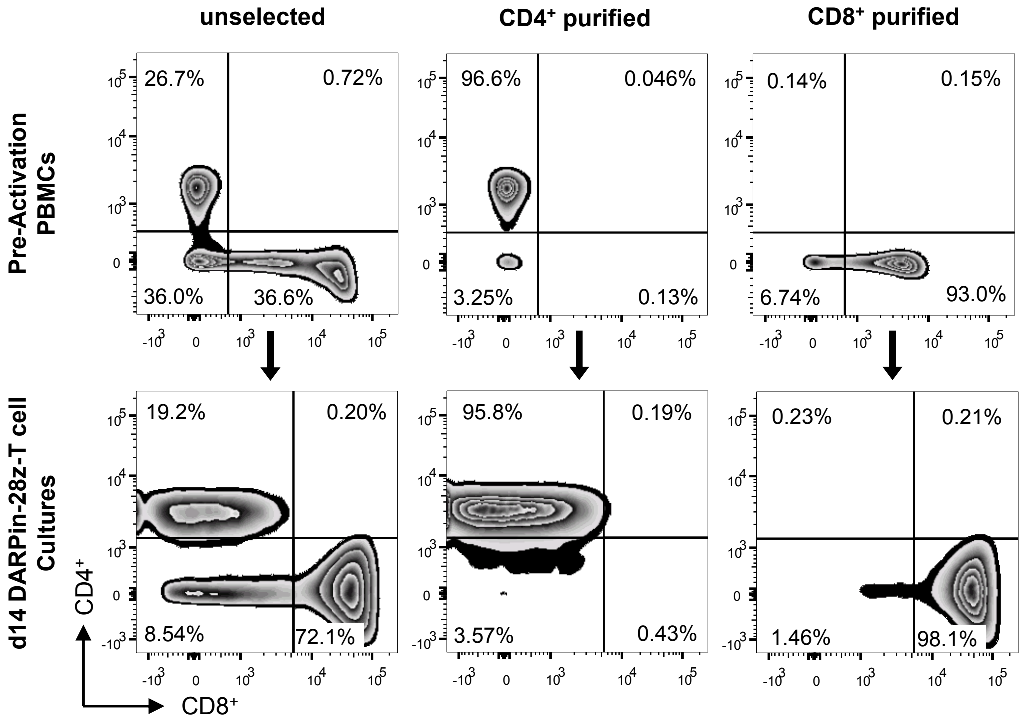

Supplemental Figure 8. Composition of CD4$^+$ and CD8$^+$ T cells in unselected vs purified DARPin-28z-T cell cultures . DARPin-28z-T cells were generated from MAC014 PBMCs that were unselected or enriched for CD4$^+$ or CD8$^+$ T cells via negative magnetic selection. Purity of CD4$^+$ and CD8$^+$ cells on day 0 (post-sort, pre-activation/engineering) and after 14 days in culture (post-activation/engineering) was assessed by flow cytometry; data representative of n=4-8 independent experiments are shown (upstream gating strategy: lymphocytes → singlets).

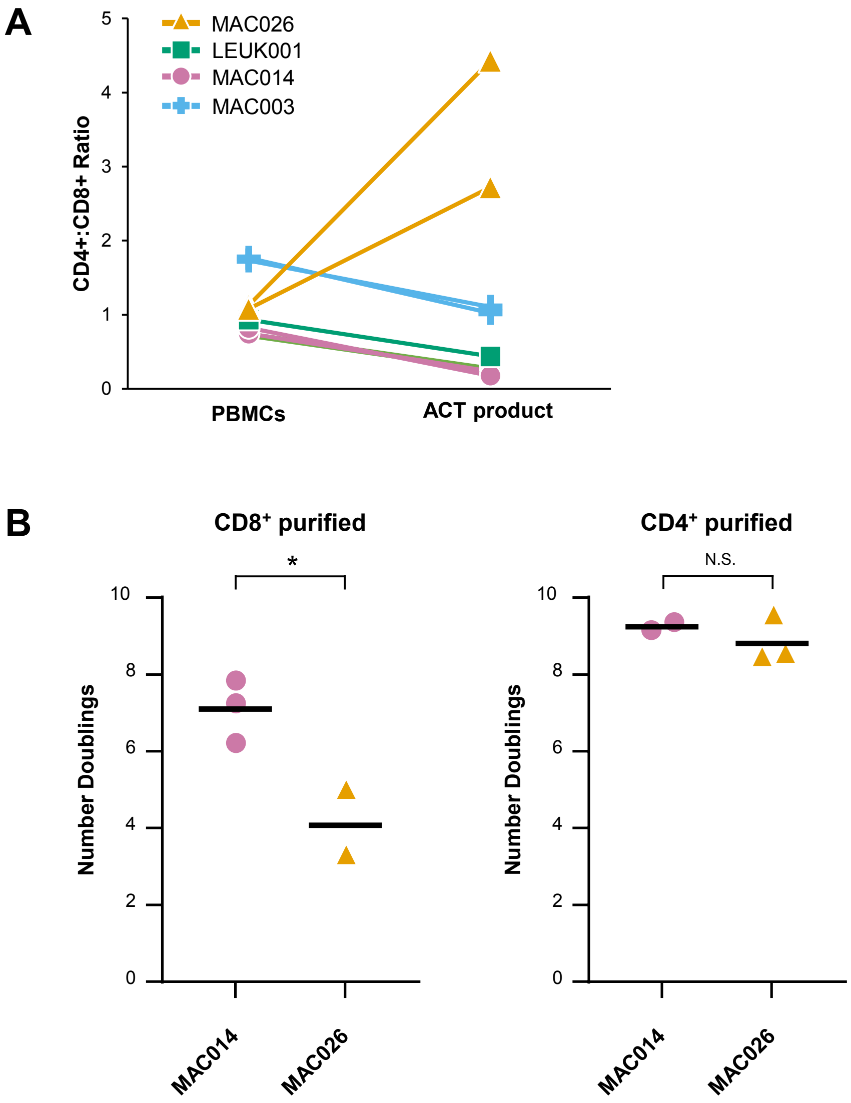

Supplemental Figure 9. Donor-specific CD4$^+$ T cell bias in the adoptive transfer product arose during *ex vivo* expansion. DARPin-28z-T cells were engineered from thawed PBMCs (various donors, as indicated (gold triangles = MAC026, teal squares = LEUK001, pink circles = MAC014, blue crosses = MAC003)) and evaluated after 14d in culture (ACT product). **A**. Freshly thawed PBMCs or the 14d DARPin-28z-T cell products they generated were stained for CD4$^+$ and CD8$^+$ and detected by flow cytometry (gating strategy: lymphocytes → singlets → CD4$^+$ vs CD8$^+$). The ratio of single-positive CD4$^+$:CD8$^+$ cells are presented. Each line indicates a single PBMC → DARPin-28z-T cell culture. **B**. Purified CD8$^+$ or CD4$^+$ T cells were generated from thawed PBMCs via negative magnetic selection and engineered to become DARPin-28z-T cells. Absolute count over time was followed and number of doublings at d14 are shown. Each point indicates expansion data from a single DARPin-28z-T cell culture (each from an independent experiment). Black lines indicate mean values.

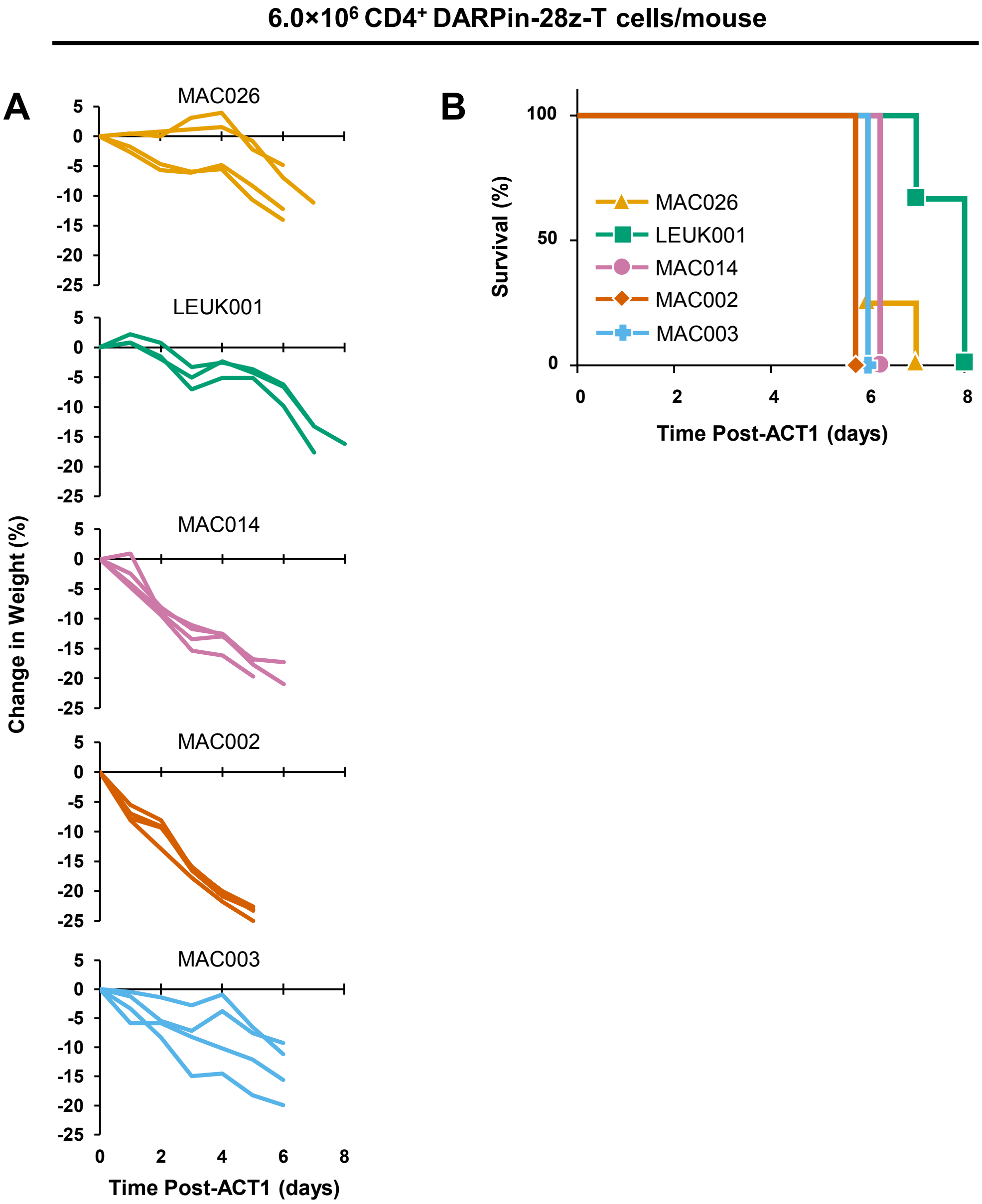

Supplemental Figure 10. CD4$^+$ purified DARPin-28z-T cells generated from a variety of PBMC donors caused similar toxicity at increased doses. Tumor-bearing NRG mice were treated with 6.0×10^6 CD4$^+$ purified DARPin-28z-T cells generated from a panel of five different PBMC donors (as indicated: gold triangles = MAC026, teal squares = LEUK001, pink circles = MAC014, orange diamonds = MAC002, blue crosses = MAC003). Mice were followed for changes in weight (**A**) (each line shows data from one mouse; n = 3-4 per donor) and survival (**B**).

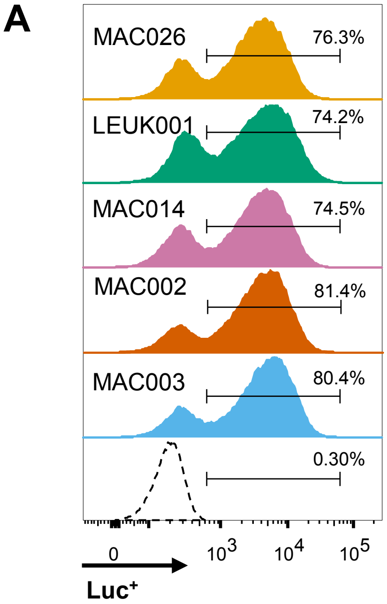

Supplemental Figure 11. DAPRin-28z-CAR-T cells generated from a five PBMC-donor panel were co-transduced with a *firefly* luciferase-expressing lentivirus to permit *in vivo* bioluminescent imaging. Purified $CD4^+$ DARPin-28z-CAR-T cells were generated from a panel of five different PBMC donors (MAC026, LEUK001, MAC014, MAC002, and MAC003); cells were co-transduced with a *firefly* luciferase-expressing lentivirus. **A**. Expression of luciferase was determined by flow cytometry (gating strategy: lymphocytes → singlets → Luc histogram). Percent Luc^+ is indicated. Dotted histogram shows a secondary only staining control. **B**. OVCAR-3 tumor-bearing NRG mice (n = 3 per treatment) received 2.0×10^6 $CD4^+$ purified DARPin-28z-CAR-T cells. After injection of D-luciferin substrate, mice were subjected to bioluminescent imaging at various time points post-ACT1 (as indicated). Images were acquired with aperture: f4, exposure: 1s. A white "X" indicates the mouse had succumbed to toxicity prior to the measurement.

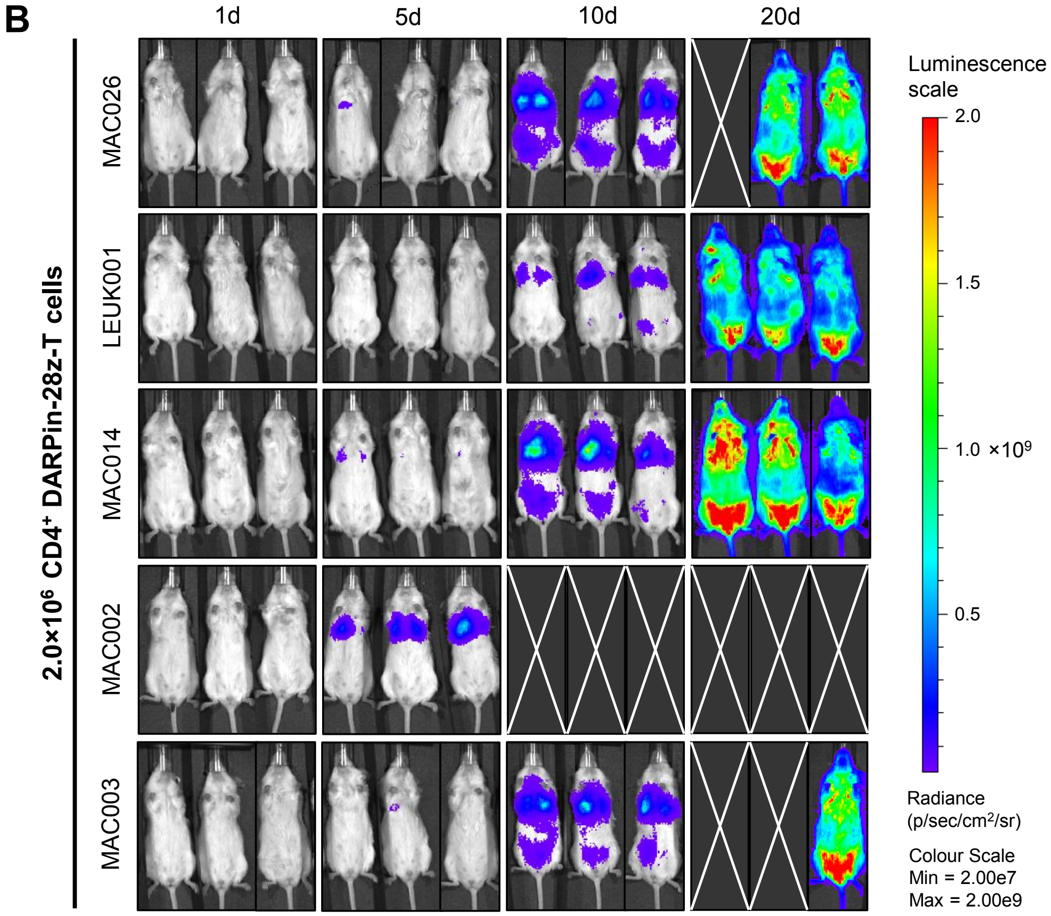

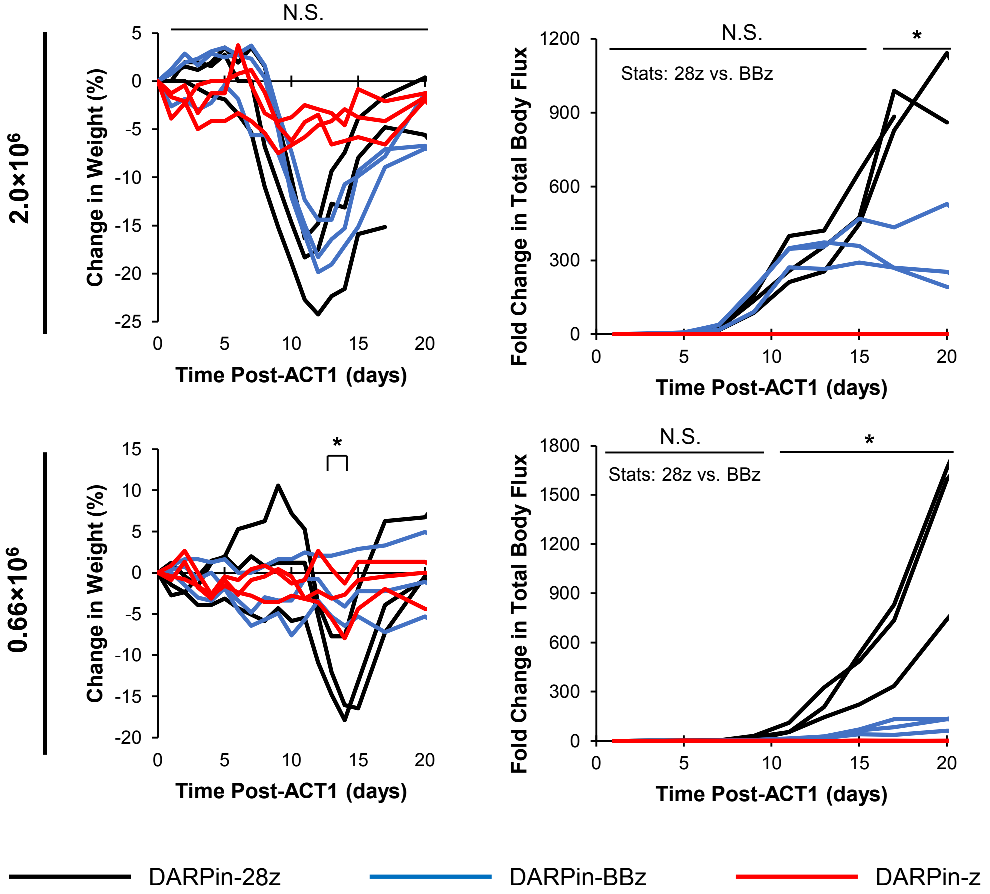

Supplemental Figure 12. Toxic hierarchy of anti-HER2 DARPin CAR scaffolds (28z > BBz > z) correlated with *in vivo* expansion. Tumor-bearing NRG mice were treated with 2.0×10^6 or 0.66×10^6 $CD4^+$ purified LEUK001 DARPin-28z- (black curves), DARPin-BBz (blue curves), or DARPin-z-T cells (red curves). All T cells had been co-transduced with *firefly* luciferase. Mice were followed for changes in weight and T cell expansion (bioluminescent imaging). Each line shows data from one mouse; curves end when mice succumbed to toxicity.

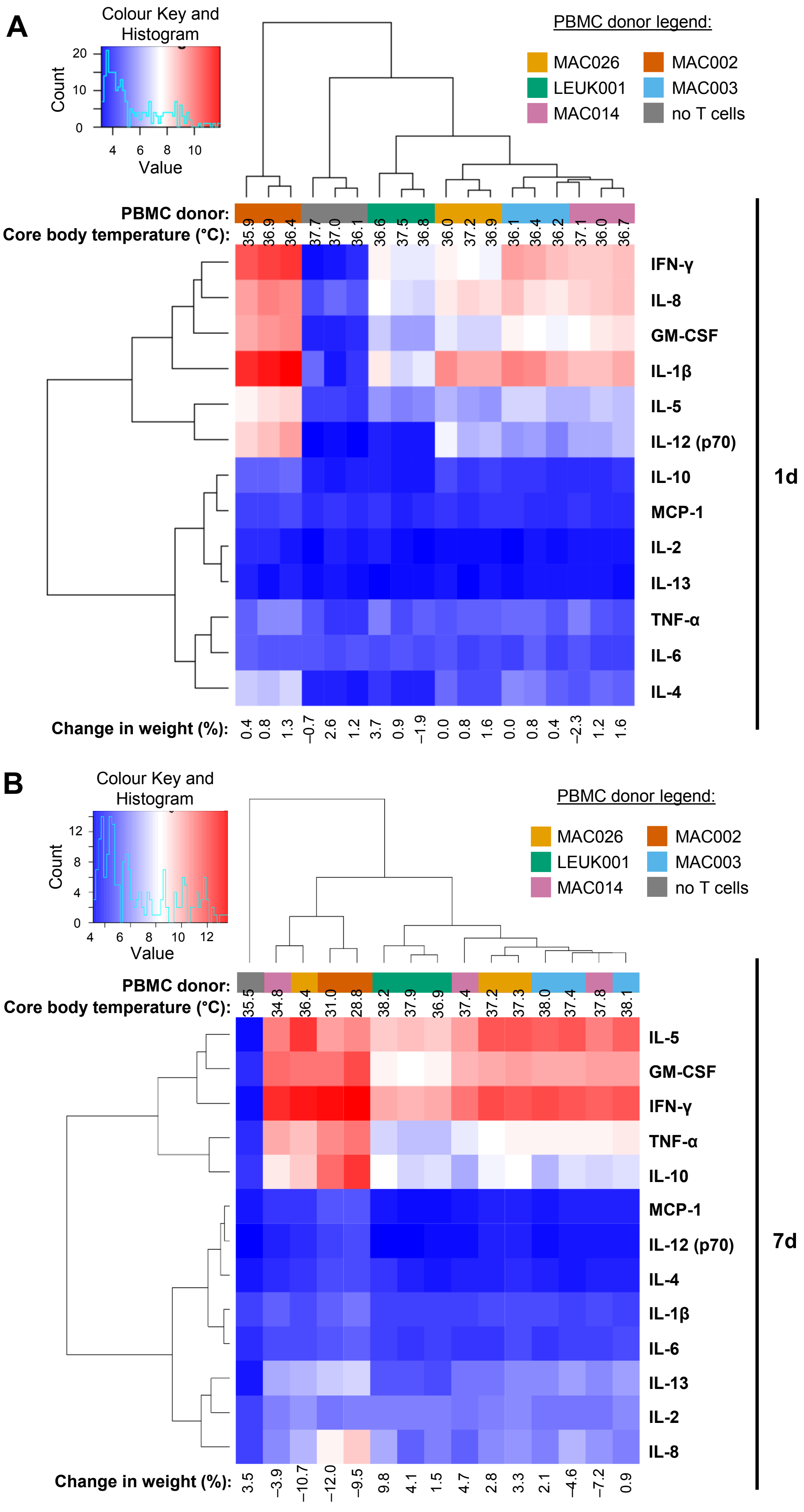

Supplemental Figure 13. Hierarchical clustering of human serum cytokine levels. Tumor-bearing NRG mice were treated with 2.0×10^6 $CD4^+$ purified DARPin-28z-T cells generated from a panel of five different PBMC donors (MAC026 (gold), LEUK001 (teal), MAC014 (pink), MAC002 (orange), and MAC003 (blue)) or no T cells (grey – vehicle only control). At one (**A**) or seven (**B**) days post-ACT1 mice were bled for multiplex analysis of human serum cytokine content (using a 13-plex panel). Log2-transformed fluorescence intensity values from the multiplex results (each being the average of n = 2 technical replicates) were analyzed through hierarchical clustering; heat maps were globally normalized, legends as shown. Change in weight (percent versus ACT1) and core body temperature (°C) at time of bleed for each mouse has been overlaid on the clustering data. Each column displays data from a single mouse; n = 3 per treatment (d1), n = 1-3 per treatment (d7).

A

	Weight Loss	Core Body Temperature
GM-CSF	0.90	-0.83
IFN-γ	0.92	-0.86
IL-1b	0.64	-0.39
IL-2	-0.03	0.07
IL-4	0.64	-0.41
IL-6	0.76	-0.78
IL-8	0.42	-0.73
IL-10	0.32	-0.48
IL-12 (p70)	0.58	-0.40
MCP-1	0.83	-0.81
TNF-α	0.48	-0.64
IL-13	0.35	-0.40
IL-5	0.30	-0.27

Supplemental Figure 14. Serum GM-CSF, IFNg, IL-6, and MCP-1 levels showed strong linear correlation with toxicity. Tumor-bearing NRG mice were treated with 2.0×10^6 or 6.0×10^6 $CD4^+$ purified DARPin-28z-T cells generated from a panel of five different PBMC donors (MAC026, LEUK001, MAC014, MAC002, and MAC003). At five (6.0×10^6) or seven (2.0×10^6) days post-ACT1 mice were bled for multiplex analysis of human serum cytokine content (using a 13-plex panel). Across all donors and both doses, the level of serum cytokine (raw fluorescence intensity value; an average of n = 2 technical replicates) was compared to severity of toxicity (as measured by weight loss or core body temperature at time of bleed) using Pearson's coefficient of correlation (r). **A**. Correlation coefficients between toxicity (weight loss *or* temperature) and serum cytokine levels are presented. For those cytokines achieving a correlation coefficient of > 0.7, data is presented graphically: **B.** GM-CSF, **C**. IFN-γ, **D**. IL-6, and **E**. MCP-1. Inset graphs display the same data broken down by dose (upper inset panel, as indicated: light purple = 2.0×10^6, dark purple = 6.0×10^6) or donor (lower inset panel, as indicated: gold triangles = MAC026, teal squares = LEUK001, pink circles = MAC014, orange diamonds = MAC002, blue crosses = MAC003). This experimental data matches that presented elsewhere in Figure 6 and Supplemental Figures 7-9.

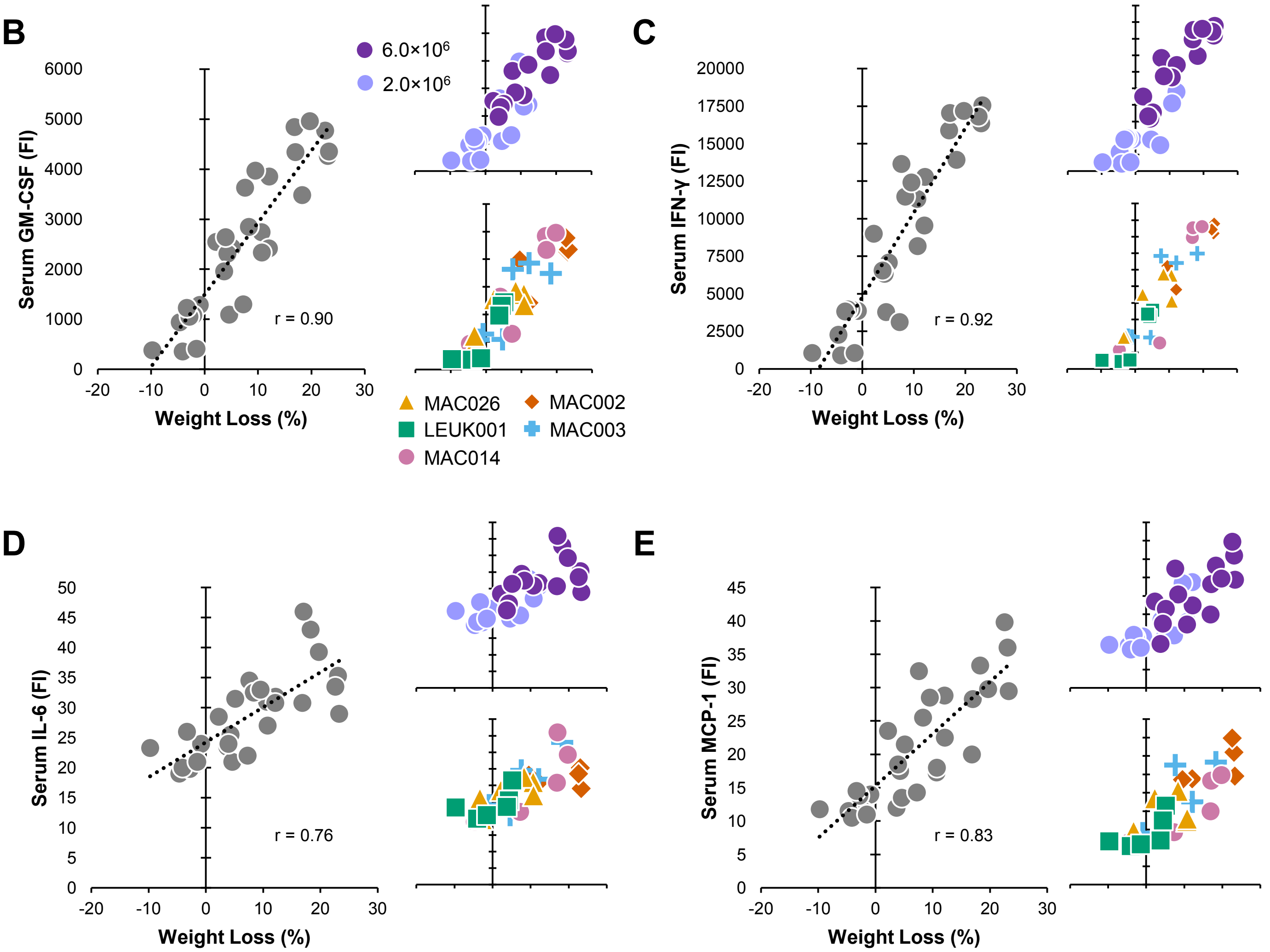

A

	OVCAR-3 stimulated						unstimulated				
	MAC002	MAC003	MAC014	MAC026	LEUK001	no T cell	MAC002	MAC003	MAC014	MAC026	LEUK001
GM-CSF	4439.1	4210.9	4179.8	2976.3	1673.9	-	46.3	22.0	21.0	15.1	67.0
IFN-γ	2937.0	1196.6	713.5	589.7	2023.4	-	40.4	12.3	8.5	13.5	155.6
IL-1β	2.6	3.8	3.3	2.1	2.4	-	-	-	-	-	-
IL-2	7318.2	6312.6	6004.7	5772.6	2215.2	-	-	-	-	-	-
IL-4	1545.6	1470.3	861.8	969.8	395.6	0.5	4.7	4.8	1.5	2.6	9.5
IL-6	1628.3	1032.2	851.2	807.1	902.6	158.0	0.2	-	-	-	0.5
IL-10	2844.4	2777.7	1935.5	3396.4	1682.1	2.5	67.8	51.6	45.1	75.2	133.3
IL-12 (p70)	0.7	0.7	0.8	0.8	0.6	-	-	-	-	-	-
MCP-1	15.7	11.9	16.3	13.4	21.6	-	-	-	-	-	-
TNF-α	5282.8	5314.2	4194.6	4416.8	3033.0	5.7	27.3	14.9	10.4	10.9	61.4
IL-13	7993.3	8028.3	5617.8	4979.0	1938.0	-	118.2	91.6	23.4	49.6	156.0
IL-5	42.8	28.1	26.0	41.8	13.5	0.1	6.7	3.9	2.7	5.8	2.8

B

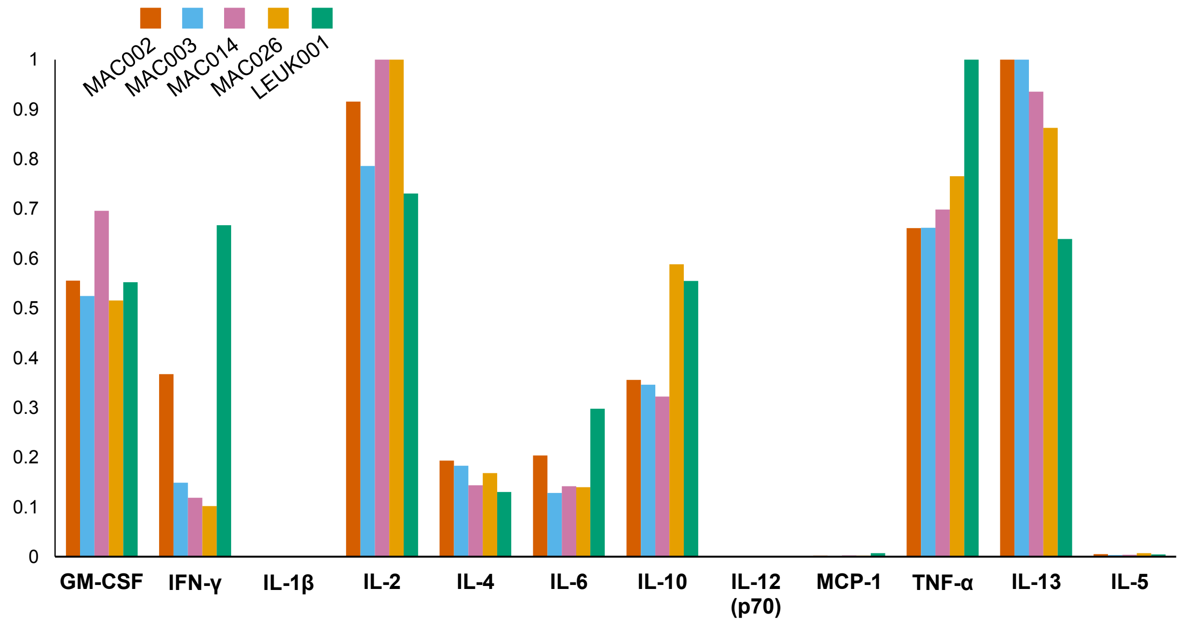

Supplemental Figure 15. *In vitro* cytokine production by CD4^{+}-purified donor-variant DARPin-28z-T cell products. Purified CD4^{+} DARPin-28z-T cells were generated from our panel of five different PBMC donors (MAC002, MAC003, MAC014, MAC026, or LEUK001). DARPin-28z-T cell products were co-cultured with tumor cell targets (HER2-positive; OVCAR-3) at a 0.5:1 (CAR-T cell to tumor cell) ratio for six hours (or in the absence of stimuli). Culture supernatants were collected for multiplex analysis of human cytokine content. **A.** Absolute cytokine concentrations in pg/mL (values are the average of 2 technical replicates). Dashes indicate values that fell below the standard curve range (not quantifiable). **B.** To reflect the relative frequencies of cytokine per donor, absolute values of cytokines (as produced by OVCAR-3-stimulated DARPin-28z-T cell products; see A.) were normalized to mode by donor.

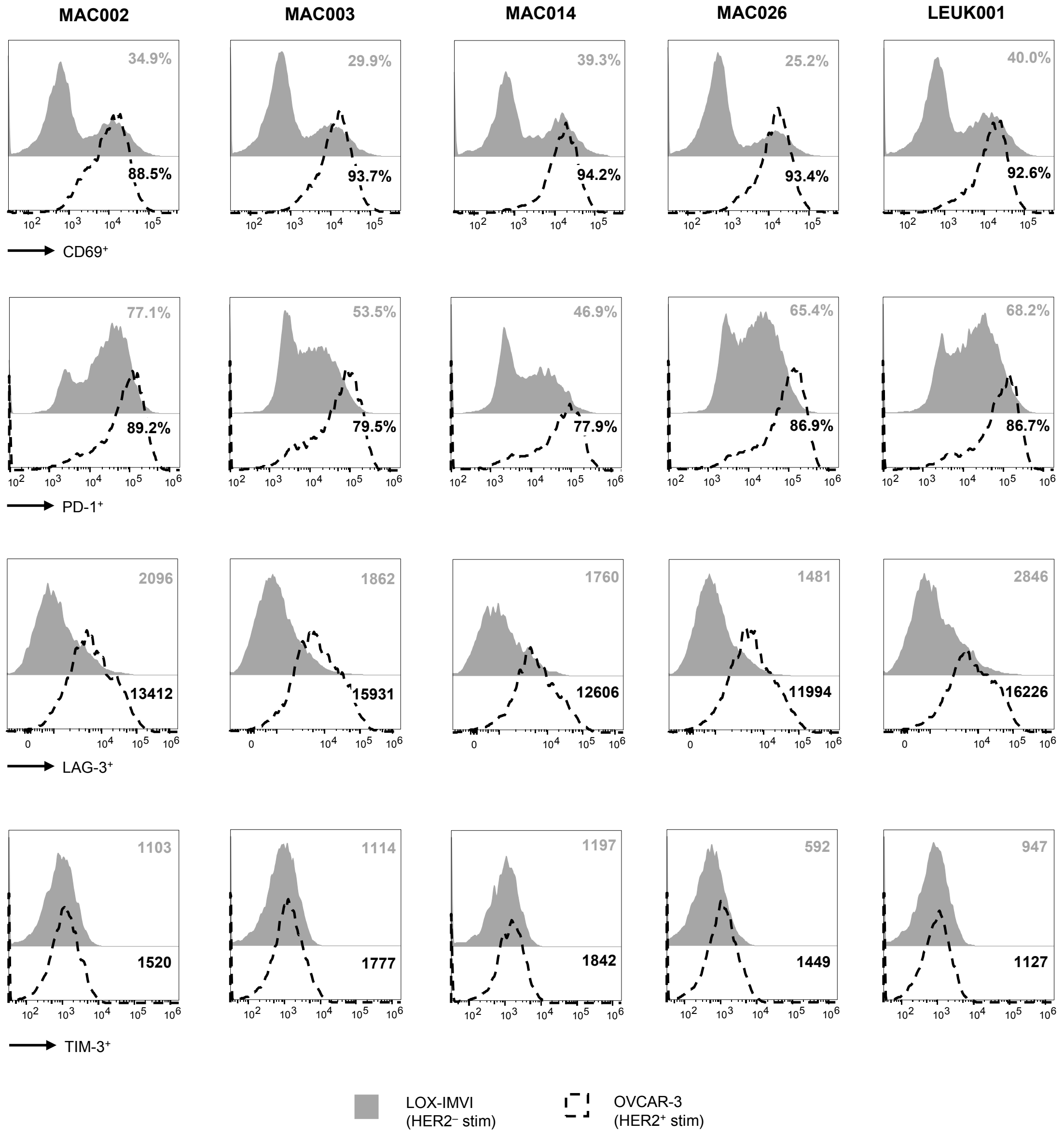

Supplemental Figure 16. DARPin-28z-T cell products showed similar rates of activation and exhaustion marker expression in response to HER2-stimulation, irrespective of donor. Cryopreserved DARPin-28z-T cell products generated from our 5-donor PBMC panel were thawed and rested for 24hrs prior to stimulation with HER2-negative (LOX-IMVI; grey histograms) or HER2-positive (OVCAR-3; dashed histograms) tumor targets. After 24hrs of co-culture, DARPin-28z-T cells were analyzed by flow cytometry for expression of activation (CD69) and exhaustion (PD-1, LAG-3, and TIM-3) markers. Numerical values quantify the frequency of target positive cells (%; CD69, PD-1) or MFI of target expression (LAG-3, TIM-3). Upstream gating: lymphocytes → singlets → live cells → $NGFR^+$ → $CD4^+$.

Supplemental Table 1. Human serum cytokine concentrations of DARPin-28z- or NGFR-T cell treated mice. OVCAR-3 tumor-bearing NRG mice were treated with 6×10^6 DARPin-28z-T cells (or an excess number of donor-matched NGFR-T cells). Mice were bled at 1, 3, 5, or 7d post-ACT1 for multiplex analysis of human serum cytokine content. Values presented are average serum cytokine concentrations (n = 3 mice, with n = 2 technical replicates/mouse) in pg/mL ± SEM.

	1d		3d		5d		7d	
	DARPin-28z	NGFR	DARPin-28z	NGFR	DARPin-28z	NGFR	DARPin-28z	NGFR
GM-CSF	803.0 (± 76.1)	3.9 (± 0.4)	1759.4 (± 120.2)	4.5 (± 0.9)	2023.1 (± 209.6)	2.0 (± 0.4)	2885.4 (± 172.0)	1.8 (± 0.3)
IFN-γ	445.4 (± 56.3)	4.7 (± 0.4)	1643.9 (± 128.0)	3.1 (± 0.2)	3587.5 (± 410.6)	0.6 (± 0.0)	9845.2 (± 873.0)	0.2 (± 0.1)
IL-1β	0.0 (± 0.0)	0.0 (± 0.0)	0.2 (± 0.2)	0.0 (± 0.0)	0.0 (± 0.0)	0.0 (± 0.0)	0.0 (± 0.0)	0.0 (± 0.0)
IL-2	487.8 (± 58.6)	0.5 (± 0.0)	860.2 (± 36.2)	0.5 (± 0.2)	80.7 (± 7.4)	0.1 (± 0.1)	10.1 (± 0.9)	0.0 (± 0.0)
IL-4	34.5 (± 4.5)	0.2 (± 0.2)	86.0 (± 2.1)	0.0 (± 0.0)	26.5 (± 1.5)	0.2 (± 0.2)	5.6 (± 1.0)	0.0 (± 0.0)
IL-6	0.0 (± 0.0)	0.0 (± 0.0)	0.1 (± 0.0)	0.0 (± 0.0)	0.1 (± 0.1)	0.0 (± 0.0)	0.5 (± 0.5)	0.0 (± 0.0)
IL-8	0.3 (± 0.1)	0.1 (± 0.1)	1.0 (± 0.3)	0.1 (± 0.1)	3.1 (± 0.4)	0.2 (± 0.1)	17.9 (± 2.8)	0.3 (± 0.3)
IL-10	361.9 (± 83.6)	3.2 (± 0.8)	842.4 (± 94.9)	3.4 (± 1.0)	352.6 (± 37.1)	4.1 (± 1.7)	445.6 (± 45.5)	3.6 (± 1.4)
IL-12 (p70)	0.4 (± 0.1)	0.1 (± 0.1)	0.8 (± 0.3)	0.3 (± 0.2)	1.0 (± 0.1)	0.2 (± 0.1)	1.0 (± 0.2)	0.1 (± 0.0)
MCP-1	0.0 (± 0.0)	0.0 (± 0.0)	0.0 (± 0.0)	0.0 (± 0.0)	0.0 (± 0.0)	0.0 (± 0.0)	0.2 (± 0.2)	0.0 (± 0.0)
TNF-α	51.2 (± 14.3)	0.7 (± 0.1)	114.0 (± 16.8)	0.6 (± 0.3)	126.1 (± 28.4)	0.4 (± 0.1)	159.1 (± 69.8)	0.2 (± 0.1)
IL-13	5.1 (± 1.2)	0.0 (± 0.0)	34.0 (± 3.3)	0.1 (± 0.1)	41.6 (± 4.2)	0.0 (± 0.0)	47.0 (± 3.8)	0.0 (± 0.0)
IL-5	1.2 (± 0.3)	0.3 (± 0.1)	173.4 (± 43.7)	0.1 (± 0.0)	1707.1 (± 248.5)	0.0 (± 0.0)	734.5 (± 17.6)	0.0 (± 0.0)

Supplemental Table 2. Murine serum cytokine concentrations of DARPin-28z- or NGFR-T cell treated mice. OVCAR-3 tumor-bearing NRG mice were treated with 6×10^{6} DARPin-28z-T cells (or an excess number of donor-matched NGFR-T cells). Mice were bled at 1, 3, 5, or 7d post-ACT1 for Multiplex analysis of murine serum cytokine content. Values presented are average serum cytokine concentrations (n = 3) in pg/mL ± SEM.

	1d		3d		5d		7d	
	**DARPin-28z**	**NGFR**	**DARPin-28z**	**NGFR**	**DARPin-28z**	**NGFR**	**DARPin-28z**	**NGFR**
**Eotaxin**	751.1 (± 19.1)	769.1 (± 41.8)	1056.3 (± 83.5)	783.3 (± 60.1)	812.4 (± 62.7)	773.6 (± 37.9)	1106.3 (± 31.8)	1002.8 (± 65.1)
**G-CSF**	701.8 (± 87.2)	561.2 (± 49.7)	3115.7 (± 1026.4)	9509.8 (± 9079.4)	10077.9 (± 1291.0)	437.3 (± 64.6)	32231.1 (± 3202.8)	332.3 (± 35.6)
**GM-CSF**	11.8 (± 2.0)	21.6 (± 1.6)	19.5 (± 2.0)	17.0 (± 3.8)	17.5 (± 1.9)	14.3 (± 1.5)	14.0 (± 1.5)	15.4 (± 2.5)
**IFN-γ**	6.1 (± 3.0)	9.0 (± 0.6)	7.3 (± 0.8)	6.1 (± 0.8)	6.0 (± 1.6)	5.4 (± 0.7)	10.5 (± 0.7)	7.8 (± 0.5)
**IL-1a**	1191.4 (± 118.3)	1237.5 (± 192.7)	1141.7 (± 60.3)	1138.9 (± 59.2)	1378.4 (± 54.4)	1459.1 (± 59.5)	1572.7 (± 51.8)	1478.2 (± 80.9)
**IL-1b**	12.9 (± 7.0)	20.1 (± 3.2)	21.3 (± 3.2)	19.8 (± 8.5)	16.0 (± 3.0)	18.9 (± 3.5)	24.6 (± 3.9)	9.6 (± 2.6)
**IL-2**	6.5 (± 2.0)	8.0 (± 2.4)	9.3 (± 1.2)	10.0 (± 3.2)	8.9 (± 1.0)	7.3 (± 1.2)	10.6 (± 1.9)	5.1 (± 2.3)
**IL-3**	1.8 (± 0.9)	2.6 (± 0.5)	1.7 (± 0.2)	2.0 (± 0.2)	2.0 (± 0.3)	1.9 (± 0.1)	2.2 (± 0.3)	1.7 (± 0.5)
**IL-4**	1.3 (± 0.3)	1.7 (± 0.3)	1.0 (± 0.1)	1.5 (± 0.4)	1.4 (± 0.2)	2.0 (± 0.4)	0.5 (± 0.0)	1.8 (± 0.5)
**IL-5**	0.2 (± 0.2)	0.0 (± 0.0)	0.8 (± 0.2)	0.0 (± 0.0)	2.7 (± 0.5)	0.0 (± 0.0)	0.0 (± 0.0)	0.0 (± 0.0)
**IL-6**	144.5 (± 32.6)	80.7 (± 35.2)	200.5 (± 57.0)	297.3 (± 284.0)	448.4 (± 56.7)	41.8 (± 19.9)	135.1 (± 8.2)	7.8 (± 0.7)
**IL-7**	1.5 (± 1.2)	3.0 (± 0.7)	3.2 (± 0.7)	2.3 (± 0.4)	3.3 (± 1.3)	2.0 (± 0.6)	3.0 (± 0.3)	2.3 (± 0.8)
**IL-9**	190.3 (± 59.8)	236.1 (± 18.9)	250.1 (± 25.2)	286.1 (± 79.1)	322.7 (± 18.6)	288.7 (± 47.7)	303.0 (± 17.4)	253.3 (± 22.3)
**IL-10**	7.2 (± 3.3)	12.3 (± 1.8)	12.0 (± 1.1)	9.3 (± 2.9)	13.6 (± 0.9)	10.5 (± 3.4)	20.3 (± 1.5)	8.5 (± 1.9)
**IL-12 (p40)**	35.7 (± 5.2)	54.1 (± 2.8)	48.0 (± 3.5)	49.9 (± 4.4)	45.4 (± 2.2)	56.0 (± 2.9)	52.3 (± 4.3)	53.6 (± 4.2)
**IL-12 (p70)**	16.1 (± 5.7)	19.3 (± 0.6)	18.8 (± 1.4)	16.7 (± 1.8)	15.1 (± 3.0)	15.1 (± 1.9)	16.7 (± 2.5)	13.7 (± 0.2)
**IL-13**	30.3 (± 3.9)	50.2 (± 5.9)	31.9 (± 2.8)	44.3 (± 3.8)	42.7 (± 2.3)	45.7 (± 2.4)	36.9 (± 3.2)	39.6 (± 5.0)
**IL-15**	29.1 (± 18.1)	26.3 (± 7.3)	38.7 (± 1.4)	17.7 (± 5.1)	17.2 (± 6.2)	15.5 (± 1.8)	21.3 (± 4.2)	22.8 (± 7.2)
**IL-17**	1.4 (± 0.8)	1.8 (± 0.2)	2.9 (± 0.3)	2.0 (± 0.5)	2.8 (± 0.4)	2.0 (± 0.3)	1.9 (± 0.1)	1.9 (± 0.1)
**IP-10**	227.7 (± 27.3)	198.9 (± 13.5)	372.7 (± 36.6)	173.5 (± 12.0)	337.6 (± 30.1)	162.3 (± 11.8)	221.7 (± 12.0)	153.0 (± 9.9)
**KC**	546.9 (± 93.3)	419.1 (± 182.8)	761.1 (± 82.8)	998.2 (± 780.1)	2894.3 (± 264.1)	191.2 (± 18.9)	674.6 (± 106.0)	200.2 (± 7.2)
**LIF**	0.5 (± 0.2)	0.4 (± 0.2)	1.0 (± 0.3)	0.3 (± 0.3)	0.1 (± 0.0)	0.5 (± 0.2)	2.0 (± 0.4)	0.4 (± 0.1)
**LIX**	14009.6 (± 4093.7)	11728.1 (± 742.5)	9272.5 (± 1223.8)	11515.6 (± 1769.9)	12795.5 (± 2570.3)	14538.9 (± 2533.6)	11172.8 (± 1869.1)	14823.7 (± 951.5)
**MCP-1**	791.6 (± 299.3)	57.1 (± 7.2)	950.6 (± 31.8)	71.5 (± 30.8)	2860.3 (± 222.0)	62.6 (± 3.5)	564.6 (± 26.8)	45.1 (± 4.1)
**M-CSF**	18.7 (± 6.1)	20.5 (± 1.1)	27.2 (± 2.3)	20.5 (± 3.3)	31.7 (± 2.5)	30.0 (± 2.3)	21.6 (± 2.0)	24.4 (± 2.2)
**MIG**	223.6 (± 21.4)	73.6 (± 2.1)	345.3 (± 127.0)	90.4 (± 6.0)	74.1 (± 7.4)	68.6 (± 3.8)	58.1 (± 5.6)	63.7 (± 6.2)
**MIP-1a**	125.6 (± 14.5)	137.1 (± 6.5)	121.9 (± 5.1)	139.3 (± 23.0)	147.3 (± 16.2)	148.9 (± 20.6)	167.1 (± 12.8)	153.7 (± 12.5)
**MIP-1b**	113.4 (± 13.0)	118.9 (± 4.8)	130.1 (± 3.3)	122.7 (± 7.3)	149.8 (± 4.4)	110.2 (± 4.4)	110.8 (± 1.2)	111.4 (± 9.4)
**MIP-2**	143.4 (± 58.0)	197.4 (± 23.4)	242.1 (± 15.1)	230.4 (± 18.8)	164.1 (± 35.7)	210.8 (± 38.8)	233.4 (± 35.1)	246.2 (± 16.9)
**RANTES**	84.3 (± 3.1)	83.3 (± 2.6)	94.8 (± 3.7)	82.7 (± 1.2)	106.6 (± 4.8)	93.4 (± 15.6)	94.6 (± 2.1)	83.2 (± 3.2)
**TNF-α**	0.8 (± 0.8)	0.3 (± 0.3)	4.7 (± 2.9)	0.0 (± 0.0)	5.9 (± 0.9)	0.0 (± 0.0)	7.2 (± 0.9)	0.8 (± 0.8)
**VEGF**	2.3 (± 0.3)	2.6 (± 0.2)	2.7 (± 0.1)	3.0 (± 0.4)	3.4 (± 0.2)	3.4 (± 0.5)	3.2 (± 0.1)	3.1 (± 0.3)

SUPPLEMENTAL MATERIALS AND METHODS

Functional avidity: 96-well, round-bottom plates (Corning, Cat No. 353077) were coated with various concentrations of recombinant human HER2-Fc (R&D Systems, Cat No. 1129-ER) diluted in PBS, overnight at 4°C. Plates were washed with cold (4°C) PBS. 5×10^5 culture day 14 T cells (which had been NGFR$^+$ purified on culture day 7) were added per well in T cell media with Brefeldin A (GolgiPlug, BD, Cat No. 555029). After 4 hours of stimulation, cells were stained as in "Functional analysis of CAR-T cells following stimulation with tumor cell lines" (see Materials and Methods in main text).

NGFR$^+$ purification: To enrich the fraction of transduced (NGFR$^+$) T cells in CAR-T cell products, and minimize variability in transduction between CAR-T cell cultures, engineered T cell products were sorted with the EasySep Human CD271 Positive Selection Kit II (STEMCELL Technologies, Cat No. 17849) on day 7 post-activation, following the manufacturer-recommended protocol.

***Tissue homogenate stimulation*:** Brain, heart, kidney, liver, and lungs were excised from a PBS-perfused, tumor-free, female NRG mouse. Tissues were mechanically disrupted, digested in a Type I Collagenase (1.5 mg/mL) + DNase I (0.2 mg/mL) solution for 1 hr at 37°C, and filtered (70 µm) to generate single cell suspensions. Engineered T cells (which had been NGFR$^+$ purified on culture day 7) were stained with CellTrace Violet (CTV; ThermoFisher, Cat: C34557) prior to co-culture with homogenates at a 1:1 ratio. After 4 days, T cell populations were evaluated by flow cytometry (gating strategy: lymphocytes → singlets → live cells → NGFR$^+$ → CD4$^+$ or CD8$^+$ → CTV histogram). Proliferation index was determined using Proliferation Fit Statistics in FCS Express v7 (De Novo Software, Pasadena, CA, USA).

Corticosteroid treatment: Dexamethasone 21-phosphate disodium salt (Sigma-Aldrich, Cat No. D1159) was reconstituted at 1 mg/mL in PBS and stored at 4°C. Stock solution was further diluted to 0.9 mg/mL with PBS and administered to mice at 9 mg/kg via intraperitoneal injection every 24 hours (beginning 24 hours post-ACT1).

In vitro DARPin-28z-T cell cytokine release profile: Culture day 14, CD4$^+$-purified DARPin-28z-T cell manufactured from our 5-donor PBMC panel (MAC002, MAC003, MAC014, MAC026, or LEUK001) were co-cultured with tumor cell targets (HER2-positive; OVCAR-3) at a 0.5:1 (CAR-T to tumor cell) ratio for six hours (or in the absence of stimuli). Co-cultures contained 50,000 CAR$^+$ T cells per well of a 96-well round-bottom plate in a total volume of 200 µL. Quantification of 13 human cytokines/chemokines in culture supernatants was performed in a multiplex assay by Eve Technologies (Cat No. HDF13, Eve Technologies Corporation, Calgary, AB) using the Bio-Plex 200 system and MILLIPLEX assay kits from Millipore.

DARPin-28z-T cell activation and exhaustion: Culture day 14, "bulk" DARPin-28z-T cells manufactured from our 5-donor PBMC panel were cryopreserved at 20 million cells/mL in CryoStor10 (STEMCELL Technologies Inc.), according to manufacturer recommendations. DAPRin-28z-T cells were thawed into cytokine-containing T cell media and rested for 24 hours. DARPin-28z-T cell cultures were rinsed and plated at a 2:1 ratio (CAR$^+$ T cells to tumor cells) on HER2-negative (LOX-IMVI) or HER2-positive (OVCAR-3) targets. After 24 hours of co-stimulation, DARPin-28z-T cells were stained for a panel of activation/exhaustion markers and analyzed by flow cytometry on a CytoFLEX LX (Beckman Coulter): Zombie NIR Fixable Viability Kit (BioLegend, Cat No. 423106), anti-CD4 AF700 (OKT4, ThermoFisher Scientific, Cat No. 56-0048-82), anti-CD8 PerCPCy5.5 (RPA-T8, ThermoFisher Scientific, Cat No. 45-0088-42), anti-PD-1 BV421 (EH12.1, BD Biosciences, Cat No. 562516), anti-LAG-3 AF647 (T47-530, BD Biosciences, Cat No. 565716), anti-TIM-3 BV785 (F38-2E2, BioLegend, Cat No. 345032), anti-CD69 BV650 (FN50, BD Biosciences, Cat No. 563835), and anti-NGFR VioBright FITC (ME20.4-1.H4, Miltenyi Biotec, 130-113-423).
